# “I Didn't Know What to Say”: Responding to Racism, Discrimination, and Microaggressions With the OWTFD Approach

**DOI:** 10.15766/mep_2374-8265.10971

**Published:** 2020-07-31

**Authors:** Sylk Sotto-Santiago, Jacqueline Mac, Francesca Duncan, Joseph Smith

**Affiliations:** 1 Assistant Professor of Medicine, Department of Medicine, Indiana University School of Medicine; Vice Chair for Faculty Affairs, Development, and Diversity, Department of Medicine, Indiana University School of Medicine; 2 Graduate Director of Faculty Affairs, Development, and Diversity, Department of Medicine, Indiana University School of Medicine; 3 Fellow, Division of Pulmonary, Critical Care, Sleep, and Occupational Health, Department of Medicine, Indiana University School of Medicine; 4 Assistant Professor of Clinical Medicine, Division of Pulmonary, Critical Care, Sleep, and Occupational Health, Department of Medicine, Indiana University School of Medicine; Statewide Internal Medicine Sub-Internship Director, Division of Pulmonary, Critical Care, Sleep, and Occupational Health, Department of Medicine, Indiana University School of Medicine

**Keywords:** Diversity, Health Equity, Inclusion, Anti-racism, Professional Development, Racism, Discrimination, Microaggressions, Faculty Development, Clinical Learning Environment, Cultural Competence, Case-Based Learning

## Abstract

**Introduction:**

Academic medicine has long faced the challenge of addressing health inequities, reflecting on how these contribute to structural racism, and perpetuating negative social determinants of health. Most recently, we have constructed opportunities for dialogues about racism, discrimination, and microaggressions (RDM). As such, we created a professional development program that encouraged participants to (1) openly discuss RDM and the impact they have in academia, (2) learn about tools to address and respond to RDM, and (3) move towards the creation of inclusive environments. The target audience included institutional leaders, faculty, trainees, professional staff, and health care teams.

**Methods:**

We sought to meet workshop goals by integrating anti-racist dramaturgical teaching, introducing concepts knowledge, and practicing communication tools. To assess learning and evaluate our workshops, participants completed a pre- and postsurvey.

**Results:**

Results showed that 30 participants were more comfortable with discussing issues related to race/ethnicity, gender identity/expression, sexual orientation, and spirituality after participating in the workshops. Prior to the two workshops, the percentage of learners who felt confident initiating conversations ranged from 29% to 54%. After the workshops, the percentage of learners who felt confident ranged from 58% to 92%. The greatest increase, 100%, was observed in the levels of confidence in initiating conversations related to race/ethnicity.

**Discussion:**

Despite medical education's commitment to cultural competence and institutional mission statements that value diversity, equity, inclusion, and justice, professional development opportunities are limited. Participants strongly agreed their participation in such a workshop was relevant and important to their professional work.

## Educational Objectives

By the end of this activity, learners will be able to:

1. Develop as allies, active bystanders, and accomplices for equity by increasing comfort and confidence in conversations associated with racism, discrimination, and microaggressions (RDM).
2. Describe concepts associated with RDM so that they can speak a common foundational language.
3. Recount instances of RDM occurring in their own educational, clinical, and professional spaces.
4. Label RDM instances so that they can minimize becoming the perpetrators.
5. Practice the OWTFD (Observe/Why?/Think/Feel/Desire) communication tool as a response tool to RDM.

## Introduction

Racism and discrimination are especially ubiquitous in our current era. The effects of racism are seen not only in health care disparities and outcomes but within academic medicine environments. In fact, academic medicine has a history of segregation, discrimination, tradition, and elitism.^1,2^ The impact of this history is visible through the recruitment, retention, and career progress of underrepresented faculty, physicians, and trainees.^3^ Unfortunately, racism, discrimination, and microaggressions (RDM) have given the racist patient a louder voice and left faculty physicians at a loss for how to respond in effective ways.^4^ We follow Smith's definition of microaggressions as (1) subtle verbal and nonverbal insults directed at people of color, often automatically or unconsciously; (2) layered insults, based on one's race, gender, class, sexuality, language, immigration status, phenotype, accent, or surname; and (3) cumulative insults, which cause unnecessary stress on people of color.^5^

Thus, all of academic medicine and especially educators need to be aware of, and discover ways to discuss and address, race and RDM.^6^ Our faculty and professional development programs are no longer spaces that can remain quiet about RDM. Relying solely on cultural competence and unconscious/implicit bias trainings is insufficient to address RDM or to promote equity and inclusion. For example, there is also great need to delve deeper into discussions that highlight the following topics within academic medicine and health care: bias, stigma and stereotyping, intercultural communication, cultural humility and reflexivity, recognizing intersectionality on health equity, privilege and power structures, and how critical race challenges equity in health care and outcomes.^7,8^ To address this gap and need, the interdisciplinary team in the Department of Medicine at the Indiana University School of Medicine developed “I Didn't Know What To Say,” a professional development program for institutional leaders, faculty, trainees, professional staff, and health care teams.

The following professional development program is anchored by anti-racist pedagogy, a paradigm that centers praxis in efforts to challenge individuals and structural systems that perpetuate racism.^9^ Praxis, which is transformation accomplished through reflection and action, requires developing critical consciousness about racism and the impacts of racism on individuals and communities.^10^ Because academic environments traditionally value objectivity and knowledge that is context independent, engaging in anti-racist work in the academy is especially challenging.^11^ Thus, all those involved (e.g., participants and facilitators) must be prepared to experience some level of emotional and mental discomfort and to disrupt the way they typically engage in professional development opportunities.

Our program is unique compared to other resources in *MedEdPORTAL* in that it explicitly focuses on microaggressions and responses to these incidents in the larger discussion of racism and discrimination.^12–19^ A number of the resources reviewed focus on important work about health care disparities, social determinants of health, cultural competence, and inclusion from the lens of curricular changes and innovation. Additionally, our program has been designed for an intended audience that spans training levels (i.e., faculty and trainees) and includes staff and administrators. Finally, our program extends an emerging approach of reenacting real case examples from one's own institution to replicate the realistic conditions under which such incidents occur.^20^

## Methods

### Development

This program was developed in 2018 by two of the curriculum's authors, Sylk Sotto-Santiago and Jacqueline Mac. The original program was constructed as a professional development series with a foundation in multiple theories and conceptual frameworks, such as critical race theory, anti-racism, and the exploration of topics including minoritization, stereotype threat, emotional labor and taxation, code-switching, racial battle fatigue, implicit/unconscious bias versus conscious inclusion, interest convergence, intersectionality, power and privilege, identity development, cultural humility, and culturally relevant pedagogies (andragogies). The session presented in this program was a truncated version that introduced the audience to fewer key concepts through the reenactment of an event experienced by another one of the authors, Francesca Duncan, involving racism. Inclusion of microaggressions was extremely important as the most salient aspect of how racism, discrimination, and bias often present.

Prerequisite knowledge by presenters and collaborators must be centered on equity. Hence, we recommend partnerships with equity, diversity, inclusion (EDI), or justice-oriented collaborators. This could be in the form of EDI experts and scholars present in offices of diversity affairs, multicultural centers, or higher education scholars. In addition, we highly encourage the workshop team to have a good sense of current sociocultural dynamics and an understanding of the experiences of underrepresented faculty and trainees, as well as sensitivity to disparities and outcomes in health care.

We sought to meet workshop goals by integrating anti-racist dramaturgical teaching. Goffman introduced dramaturgical theory by examining social interactions and using the analogy of a stage.^21^ He described this approach as viewing a social situation as a scene and people as actors who are negotiating through human interaction. More specifically, Goffman described the front stage as how society expects people to present themselves in a certain way; when a person goes against the norm, society tends to notice. Through this, we explored what the norm in society was in relation to RDM. The back stage posits the area where one's presence is no longer seen by society, where people can be themselves. Again, in the context of this reenactment, this was how the actors and participants became vulnerable and fully open about the impact of RDM in their lives. Dramaturgy aligned with experiential learning and drove a more-holistic educational development experience.

### Implementation

To accommodate all learners, we conducted two workshops. The recommended number of participants depended on departmental climates and openness to EDI initiatives. There was no ideal number, but a minimum of six to 18 per session was effective. The optimal length of each workshop was 2 hours. However, should presenters wish to include additional concepts, we recommend additional time. The optimal length took into account the process of ensuring that a brave space was nurtured and trust built. Users should view Appendix A for the workshop agenda.

Each workshop took place in a large-group setting during or shortly after lunch hour. Food and refreshments were provided. For the first 15 minutes of the session, participants entered the room, obtained lunch and refreshments, and completed the presurvey on learners' prior experiences with discussions connected to diversity-related topics, discriminatory behavior, and trainings (Appendix B). Then, we introduced the workshop by setting community agreements for supporting a brave space and providing an overview of the agenda using the PowerPoint (Appendix C).

The first half of the workshop introduced and defined racism and discrimination by engaging participants in a discussion. Racism and discrimination should be introduced as the core concepts, but this workshop may be tailored to other communities and include additional topics by consulting supplemental resources (Appendix D).

Following the introduction, we spent 30 minutes reviewing and discussing a local scenario (Appendix E). The script was about an incident that occurred in our community and was generated and presented by two guests, Francesca Duncan and Joseph Smith. They invited a third guest to play the patient role (see Appendix E for notes about the development of this script and guidance for choosing a local example of RDM for reenactment). Participants were asked to listen while the script was read, take a deep breath, and spend about 30 seconds in silence to reflect after the reenactment. Next, the two guests were asked to share their reflections (Appendix F), and workshop leaders facilitated a discussion between the participants and guests.

Before transitioning to the second half of the workshop, participants participated in an individual 10-minute reflection exercise (Appendix G) to start shifting their mindsets toward responding to such incidents. We introduced the reflection exercise as part of our efforts at situating participants in active roles. Reflection allowed for retrospection and improvement of one's actions, abilities, and knowledge.

The second half of the workshop focused on discussing microaggressions, microresistance, and possible communication tools to respond to such incidents. One such tool was the OWTFD approach, which stands for Observe/Why?/Think/Feel/Desire (see Appendix C, slide 22). After we provided an overview and discussion about the topics, pairs of participants engaged in the practice and discussion of two scenarios (Appendix C). Facilitators asked volunteer participants to share their responses. Following the practice, facilitators debriefed the workshop in the larger group with a few questions and offered additional individual or small-group debrief time, as all participants completed the postsurvey (Appendix B). The pre- and postsurveys were administered via paper and entered into Qualtrics for data analysis.

### Assessment

The overarching goal of this workshop was to increase learner confidence and comfort so as to minimize the likelihood of becoming perpetrators and to respond to incidents when they arise. We sought to achieve this goal by integrating learning components aimed at developing learner awareness, introducing concepts related to RDM, and practicing communication tools. We assessed whether the workshop positively impacted learners' level of comfort in discussing diversity-related topics with colleagues and trainees by gauging their comfort levels in the pre- and postsurveys (Appendix B). Relatedly, we also assessed whether the workshop positively impacted trainees' level of confidence with initiating conversations on diversity-related topics with colleagues and trainees. We did not match the pre- and postsurvey responses; therefore, we cannot discuss assessments that occurred at the individual level. Rather, we assessed learning from a group level.

Next, we assessed how learners evaluated the overall workshop using the Department of Medicine's standard faculty development workshop survey. Not all questions were immediately relevant to this particular workshop, so we only reviewed responses to the most-relevant questions.

The reflection exercise (Appendix G) during the workshop provided the opportunity for qualitative analysis based on two questions: (1) What motivates you to respond (or not) to instances of racism, discrimination, and/or microaggressions? (2) To what extent do you believe your role as an educator (staff or learner) influences the broader learning environment for learners? Through open and thematic coding, two investigators independently identified themes in these reflections and then came together to achieve consensus and generated collective themes. The combination of survey findings and themes generated from written reflections forms the basis of our results and subsequent discussion.

## Results

### Facilitator Characteristics

This workshop was facilitated by two individuals with collective training and expertise in equity and inclusion topics, faculty development, critical/difficult conversations, and academic medicine and health care. There were two additional cofacilitators who were willing to share their experiences of racial discrimination and responding to such an incident within a health care setting. As a team, the facilitators were self-aware and reflective individuals who approached this workshop with thoughtfulness and care. We highlight these characteristics to emphasize the importance of experience, skill, and intentionality in crafting and implementing a workshop on a difficult topic that ultimately positively impacted the learners.

### Learner Characteristics

A total of 30 learners, including faculty, professional staff, residents, fellows, and medical students, participated in two different sessions of this workshop. These individuals represented a number of different divisions within the Department of Medicine, including the Division of Family Medicine and the Division of Health Systems.

Presurvey responses (*n* = 24) indicated that learners seldom had opportunities to discuss the four diversity-related topics (i.e., race or ethnicity; gender, gender identity, or gender expression; sexual orientation; and spirituality and faith). When asked how often learners had an opportunity to discuss these topics with colleagues, more than half of learners responded with *often* or *sometimes* to the topic of race or ethnicity. However, for the remaining topics, more than half of the respondents selected *seldom* or *never.*

Even though learners seldom had the opportunity to discuss such topics, they often thought about discriminatory behavior. About 30% of the respondents indicated they thought about discriminatory behavior related to their work on a daily basis, nearly 40% on a weekly basis, and the remaining respondents monthly or every once in a while. A vast majority of learners had witnessed and/or personally experienced discriminatory behavior. Nearly 80% of respondents had witnessed discriminatory behavior in academic medicine, with about 12% who were unsure if they had witnessed such behavior. Nearly 60% of respondents had personally experienced such behavior. Many learners had participated in trainings that could support their ability to respond to such incidences. Half of the respondents had completed difficult, crucial, or courageous conversations training, 42% had completed implicit or unconscious bias training, and about 37% had completed cultural competence or humility training.

### Pre- and Postsurvey Evaluation of Impact

**Figure 1.**
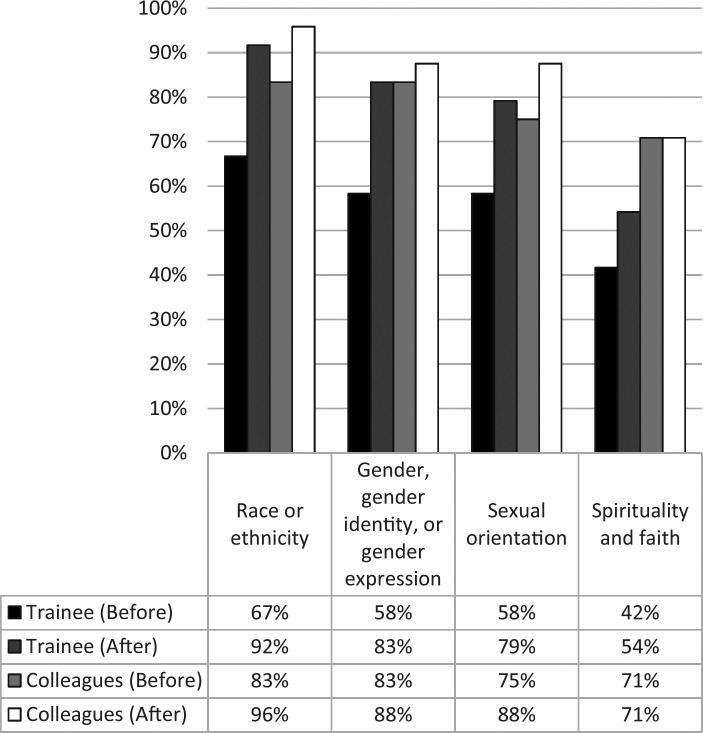
Responses to how comfortable participants felt discussing topics with colleagues or trainees. The figure shows the combined percentage of respondents (*n* = 24) who selected either *very comfortable* or *somewhat comfortable* for the question “Currently, how comfortable do you feel discussing topics with [colleagues or trainees]?” before and after the workshop.

Figure 1 displays the combined percentage of respondents (*n* = 24) who selected either *very comfortable* or *somewhat comfortable* for the question “Currently, how comfortable do you feel discussing topics with [colleagues or trainees]?” before and after the workshop. Learners, before and after the workshop, reported the highest levels of comfort with race- or ethnicity-related discussions. Prior to the workshop, the percentage of learners who felt very comfortable or somewhat comfortable discussing the four diversity-related topics with colleagues or trainees ranged from 42% to 83%. After the workshop, the percentage of learners who felt similarly ranged from 54% to 96%. Overall, the percentage of learners who selected these responses increased in the postsurvey for all four diversity-related topics. The greatest percentage increase (25%) was observed in the trainees' reported comfort levels for discussing both race or ethnicity topics and gender, gender identity, or gender expression topics.

**Figure 2.**
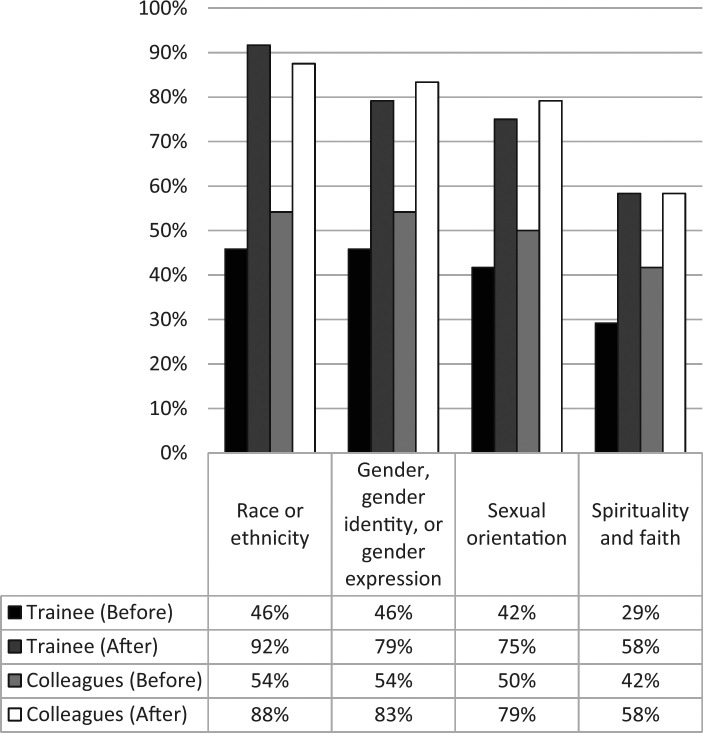
Responses to how comfortable participants felt discussing topics with colleagues or trainees. The figure shows the combined percentage of respondents (*n* = 24) who selected *very confident* or *somewhat confident* for the question “Currently, how confident do you feel initiating conversations about the following topics with [colleagues or trainees]?” before and after the workshop.

Figure 2 displays the combined percentage of respondents (*n* = 24) who selected *very confident* or *somewhat confident* for the question “Currently, how confident do you feel initiating conversations about the following topics with [colleagues or trainees]?” before and after the workshop. Learners, before and after the workshop, reported highest levels of confidence initiating conversations related to race or ethnicity, or gender, gender identity, or gender expression. Prior to the workshop, the percentage of learners who felt very confident or somewhat confident initiating conversations with trainees or colleagues on the four diversity-related topics ranged from 29% to 54%. After the workshop, the percentage of learners who felt this way ranged from 58% to 92%. The greatest percentage increase (100%) was observed in the level of confidence learners reported in initiating conversations related to race or ethnicity with trainees, which increased from 46% to 92%.

### Faculty Development Survey Evaluation of Impact

**Figure 3.**
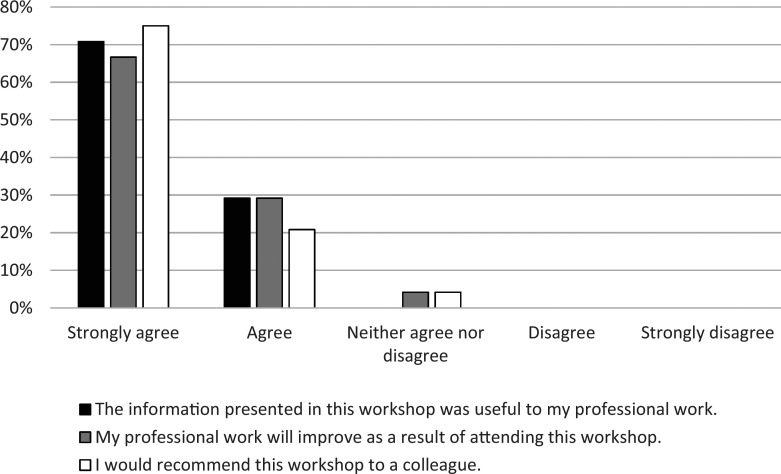
Level of agreement with evaluation statements (*n* = 24).

The overall workshop evaluation was positive. Figure 3 displays respondents' level of agreement with three items from the standard Department of Medicine faculty development survey used at the Indiana University School of Medicine. All respondents (*n* = 24) agreed or strongly agreed that the information presented was useful in their professional work. All but one respondent (*n* = 23) agreed or strongly agreed that their professional work would improve as a result of attending the workshop. Similarly, all but one respondent (*n* = 23) agreed or strongly agreed that they would recommend this workshop to a colleague.

All but one learner reported that they were at least slightly familiar with the material presented in the workshop. About half of these learners were very or extremely familiar with the materials. Even so, nearly two in three learners reported learning a great deal of new information. Many respondents shared that they would incorporate the specific communication tool practiced into their work, while others felt more empowered to “start conversations with physicians in the workplace,” “take a minute to speak to overt racism or microaggression,” and “improve curriculum and faculty development.” Learners largely shared two components of the workshop that resonated with them: the reenactment of the racial discrimination experienced and the tools shared and ability to practice those tools. Moreover, a few learners shared how this workshop validated their experiences of microaggressions and discrimination.

### Reflection Exercise

Twenty-five participants submitted their written reflections. Responders were asked, “What motivates you to respond (or not) to instances of racism, discrimination and/or microaggressions?” Their responses for why to respond fell into the following themes: (1) for others, (2) upbringing, (3) morals/values, (4) equality/equity, (5) rights/justice, (6) previous experience, (7) education, (8) agency, (9) emotional harm and individual effect, (10) protection, (11) promote change, and (12) ignorance/desensitize. Reasons for not responding included (1) power dynamics, (2) relationship, (3) time, (4) lack of knowledge on how to tackle issue, and (5) tired (racial battle fatigue). Respondents were also asked, “To what extent do you believe your role as an educator (staff or learner) influences the broader learning environment for learners?” The themes of their responses included (1) environment change/safe environment, (2) protector, (3) communicator/open conversations, (4) duty and responsibility, (5) role model, (6) inclusive, and (7) professionalism.

## Discussion

The development and implementation of this type of program are rewarding, but they can also be taxing. We would like to acknowledge that our own personal and professional lives have been impacted by experiencing these instances of RDM and/or witnessing them from colleagues, trainees, and patients. When it comes to covert forms, such as microaggressions, we have all been in a position in which we did not know what to say. In fact, “I didn't know what to say” is an all too common response. The willingness of presenters to be as vulnerable as the audience was an essential element of success. We offered this program knowing that it could be of great benefit to our academic community. The reenacted experience of responding as a trainee and a clinician-educator to a patient's racist verbal assault provided a vivid reality from which facilitators could begin an open discussion with learners. The experience demonstrated the importance of creating a safe, brave space to openly discuss this topic, which required facilitator and audience vulnerability.

Two significant assessment findings are worth further discussion. First, we found that even though the content of the workshop largely focused on race or ethnicity, learners reported increased levels of comfort in having these discussions with trainees and colleagues across other diversity-related topics. A similar pattern of increase with regard to level of confidence in initiating these conversations was also found. These findings suggest that starting the conversation of how to respond to instances of RDM can contribute to responding to instances of other types of exclusion. These findings also support the promise of subsequent follow-up trainings or, ideally, a longer-term training experience that includes other topics and opportunities to continue practicing and reflecting.

Second, learners generally reported similar levels of confidence and comfort discussing these topics with trainees and colleagues on the postsurvey, while learners reported lower levels of confidence and comfort discussing these topics with trainees compared to colleagues on the presurvey. Perhaps the use of real-life examples that demonstrated responses to instances of RDM between attending and trainee, faculty and learner, and other pairings contributed to this finding. This suggests two important implications. First, there may be a perceived or real layer of complexity to being an educator and working with a trainee when responding to instances of RDM. While this complexity is not a focal point of the training, it should be further explored. Second, such trainings are likely applicable in a myriad of settings, and future trainings should aim to include a variety of examples and cases.

It is important to explore what motivates individuals to respond to these instances of RDM. We found three overarching themes: upbringing and previous experiences; values centered on equity, equality, and justice; and agency. Specifically, our lived experiences, values compass, and courage to act are what motivates us to become active bystanders. This aligns with bystander motivation literature, which, in cases of bullying, has shown five motivators: interpretation of harm in the situation, emotional reactions, social evaluating, moral evaluating, and intervention self-efficacy.^22^ Simultaneously, we examined the reasons that prevent action: power positioning and dynamic, lack of knowledge or personal experience with the issue, and racial battle fatigue, a salient theme amongst attendees of color. Racial battle fatigue, as Smith argued, is the stress associated with racial microaggressions that causes various forms of mental, emotional, and physical strain.^5^

Despite medical education's commitment to cultural competence and institutional mission statements that value diversity, equity, inclusion, and justice, curriculum innovation and professional development opportunities are hard to implement. There are qualitative differences between the experiences of underrepresented faculty and students compared to those from majority groups in medicine. As educators, we have to acknowledge that our actions and inactions are often modeled. We were pleased to see our educators took ownership and responsibility for the learning environments. Qualitative answers highlighted the impact of our roles with these themes: promoting change, providing safe environments, the importance of critical conversations, role modeling, and professionalism.

Generalization in qualitative research is contextualized as the broad understanding of the human experience. Here, that human experience is RDM, and as such, the opportunity for generalizability is present at every higher education and academic medicine institution. As one of the aspects of generalizability, transferability to other institutions is also applicable. First, professional development programs are now a mainstay in academic medicine. Second, faculty roles have evolved along with medical education, health care systems, and society at large. Hence, faculty have to adapt in multiple ways, including teaching in complex learning environments with complex societal implications. Third, as academic medicine continues to strive to eliminate health care disparities, dismantle structural racism in health care, and address the social determinants of health, EDI and justice must be valued and serve as the foundation. Thus, all of these concepts serve as the fundamental pieces that can enable successful implementation of this program. These pieces provide a common space, common language, and, unfortunately, active-present examples.

We caution our readers to take into account institutional and departmental cultures and climates to determine their appropriate approach for local audience cohorts. Attitudinal assessments and the creation of not only safe but brave spaces are critical for having an effective and productive conversation. In addition, educators must be prepared to address the resistance, emotional responses, and vulnerability that arise from the audience and from their own experiences. Our role as faculty developers in responding to RDM relied not on supplying answers or changes in ideologies but on providing spaces that deconstructed the racism and discrimination around us, presenting informed perspectives from marginalized groups, and offering tools to support faculty in working towards inclusive environments.

Workshop-style faculty and professional development is the most common form of initiative because of its flexibility and active-learning approach.^23^ In addressing limitations of this program, we must restate our first approach of a longitudinal or fellowship-style program as a possible best approach to this content.

Given the impact of this training as evidenced by the assessment results, it behooves us to reiterate the importance of truly delving into all associated frameworks and concepts that could also provide the foundation for deeper conversations around cultural humility and structural cultural competence. The immediate impact would be in more culturally relevant andragogies, equity-centered patient care, and inclusive excellent environments. Lastly, broader implementation of such programs depends not only on expertise and experience but on institutional climates that support equity and inclusion efforts at all levels. These limitations can be addressed through the intentional delivery of concepts most relevant to the institution's climate, the demographics of the institution, and the local population. In addition, current and successful professional development programs present at the institution may determine the type of format that would work best there. Deeper conversations require courage and trust and can only be guided by expertise. Good intentions without expertise or experience can cause greater damage to a fragile conversation. It is critical to partner with individuals who have the expertise and can complement each other's skill set.

Future programming and research should focus on culturally relevant environments in medical education and continue to question institutional commitments to equity. Future directions still include the necessity for fellowship-style programming that enhances equity, inclusion, and justice work while also embracing educational development and culturally relevant andragogy and honoring the missions of our institutions. In addition, for those academic medicine centers associated with health care or hospital systems, we need to develop better alignment with policies, procedures, and engagement of the entire health care workforce. The scope of this work should not rely on minoritized members of our academic communities.

To understand the full impact of this program, one must understand the experiences of marginalized and underrepresented groups in medicine within the current US sociopolitical environment and within a technological environment that allows more overt and covert instances of RDM to occur with some amount of anonymity and distance. It is within this urgent context that we hoped to provide a program that could bring awareness to these experiences, as well as tools to counter them in academic medicine. With this program, we have been able to provide an inclusive community space allowing reflection on and evaluation of our own role as educators and as members of the academy.

## Appendices

Workshop Agenda.docxPre- and Postsurvey.docxI Didn't Know What to Say.pptxSupplemental References.docxScenario Reenactment Script.docxScenario Guest Reflections.docxReflection Exercise.docx
All appendices are peer reviewed as integral parts of the Original Publication.
