# Supplementary material for: “I Didn't Know What to Say”: Responding to Racism, Discrimination, and Microaggressions With the OWTFD Approach
Source: MedEdPORTAL. 2020 Jul 31;16:10971. doi: 10.15766/mep_2374-8265.10971 (PMC7394349; doi:10.15766/mep_2374-8265.10971)
Supplement: Supplementary file 1 — Workshop Agenda.docxPre- and Postsurvey.docxI Didn't Know What to Say.pptxSupplemental References.docxScenario Reenactment Script.docxScenario Guest Reflections.docxReflection Exercise.docx [file mep_2374-8265.10971-s001.zip › A. Workshop Agenda.docx]

**Appendix A. Workshop Agenda (Playbook)**

| Timing | Activity | Materials |
| --- | --- | --- |
| 15 Minutes | **Welcoming Participants**  Recommended number of participants depends on departmental climates and openness to DEI initiatives. There is no ideal number, but a minimum of 6-18 per session.  Target: institutional leaders, faculty, trainees, professional staff, and healthcare teams   1. Welcome participants 2. Participants completing pre-assessment | - Copies of pre-assessment (Appendix B) - Pens |
| 5 Minutes | **Introductions and Setting the Space**   1. Introduce workshop 2. Review pre-generated community agreements and define brave space. Some questions to ask:    1. *What* *clarifying questions or comments do you have about these agreements?*    2. *Are there other agreements you’d like to add to this list?* 3. Overview of agenda | - PowerPoint Slides 1-5 (Appendix C) |
| 30 Minutes | **Racism and Discrimination**   1. Overview definitions and how these concepts show up in health care. 2. Share scenarios (slides 11 and 12) or reenact an incident that occurred in your community. 3. Ask participants to take some deep breaths, and 30-60 seconds of silent reflection. 4. Debrief scenarios. Suggested questions to ask participants:    1. *What thoughts crossed your mind as you witnessed (or read) these scenarios?*    2. *If reenactment, what questions do you have for our colleagues?* | - PowerPoint Slides 6-12 (Appendix C) - Scenarios and/or script (Appendix E) - Guest scenario reflections (Appendix F) |
| 10 Minutes | **Individual Reflection**   - Distribute copies of reflection exercise and ask participants to silently reflect on their own. | - Copies of reflection exercise (Appendix G) - Pens |
| 20 Minutes | **Microaggressions, Micro Resistance, and Communication Tools**   1. Define microaggression 2. Offer time for a Q&A for participants to wrestle with, unpack, clarify the distinctions between racism, discrimination, and microaggressions 3. Introduce the concept of micro resistance 4. Overview a few communication tools 5. *Note: Slides 22-25 are other tools. Feel free to practice multiple tools or include ones that your community is familiar with.* 6. Demonstrate OW*TFD with a scenario relevant to your community. Co-facilitators should role play to demonstrate how to respond. | - PowerPoint Slides 13-25 (Appendix C) |
| 20 Minutes*  * This is the minimum amount of time. Allow at least 7 minutes per pair per scenario. | **Practicing Responses**   - Divide participants into pairs to practice scenarios on Slides 28 and 29.   - *Note: Encourage participants to talk about the scenario, bring up similar scenarios, or ask each other questions.* - Allow 5-10 minutes for volunteer participants to share/demonstrate how they responded to each scenario. | - PowerPoint Slides 26-29 (Appendix C) |
| 20 Minutes | **Workshop Debrief and post-assessment**   1. Debrief participants’ experience of the workshop. Suggested questions are on slide 30. Offer additional time to debrief after the workshop. 2. Distribute post-assessment | - PowerPoint Slides 30-31 (Appendix C) - Copies of post-assessment (Appendix B) - Pens |
| ***30 Minutes*** | ***Optional additional debrief time*** |  |
