# Supplementary material for: “I Didn't Know What to Say”: Responding to Racism, Discrimination, and Microaggressions With the OWTFD Approach
Source: MedEdPORTAL. 2020 Jul 31;16:10971. doi: 10.15766/mep_2374-8265.10971 (PMC7394349; doi:10.15766/mep_2374-8265.10971)
Supplement: Supplementary file 1 — Workshop Agenda.docxPre- and Postsurvey.docxI Didn't Know What to Say.pptxSupplemental References.docxScenario Reenactment Script.docxScenario Guest Reflections.docxReflection Exercise.docx [file mep_2374-8265.10971-s001.zip › B. Pre- and Postsurvey.docx]

**Appendix B. Pre and Postsurvey**

**Responding to Instances of Racism, Discrimination, and Microaggressions Pre-Survey**

*Thank you for registering for this workshop. Please take about* ***5 minutes*** *to complete this presurvey. This information will be used to tailor the design of the workshop and to evaluate the effectiveness of this workshop.*

1. Currently, how **comfortable** do you feel discussing the following topics with colleagues?

|  | Very comfortable | Somewhat comfortable | Somewhat uncomfortable | Very uncomfortable |
| --- | --- | --- | --- | --- |
| Race or ethnicity | ○ | ○ | ○ | ○ |
| Gender, gender identity, or gender expression | ○ | ○ | ○ | ○ |
| Sexual orientation | ○ | ○ | ○ | ○ |
| Spirituality and faith | ○ | ○ | ○ | ○ |

1. Currently, how **comfortable** do you feel discussing the following topics with trainees?

|  | Very comfortable | Somewhat comfortable | Somewhat uncomfortable | Very uncomfortable |
| --- | --- | --- | --- | --- |
| Race or ethnicity | ○ | ○ | ○ | ○ |
| Gender, gender identity, or gender expression | ○ | ○ | ○ | ○ |
| Sexual orientation | ○ | ○ | ○ | ○ |
| Spirituality and faith | ○ | ○ | ○ | ○ |

1. Currently, how **confident** do you feel initiating conversations about the following topics with colleagues?

|  | Very confident | Somewhat confident | Somewhat not confident | Not confident at all |
| --- | --- | --- | --- | --- |
| Race or ethnicity | ○ | ○ | ○ | ○ |
| Gender, gender identity, or gender expression | ○ | ○ | ○ | ○ |
| Sexual orientation | ○ | ○ | ○ | ○ |
| Spirituality and faith | ○ | ○ | ○ | ○ |

1. Currently, how **confident** do you feel initiating conversations about the following topics with trainees?

|  | Very confident | Somewhat confident | Somewhat not confident | Not confident at all |
| --- | --- | --- | --- | --- |
| Race or ethnicity | ○ | ○ | ○ | ○ |
| Gender, gender identity, or gender expression | ○ | ○ | ○ | ○ |
| Sexual orientation | ○ | ○ | ○ | ○ |
| Spirituality and faith | ○ | ○ | ○ | ○ |

**TURN OVER 🡪**

1. How often do you have an opportunity to discuss the following topics with your colleagues as it relates to your work?

|  | Often | Sometimes | Seldom | Never |
| --- | --- | --- | --- | --- |
| Race or ethnicity | ○ | ○ | ○ | ○ |
| Gender, gender identity, or gender expression | ○ | ○ | ○ | ○ |
| Sexual orientation | ○ | ○ | ○ | ○ |
| Spirituality and faith | ○ | ○ | ○ | ○ |

1. Have you witnessed discriminatory behavior in academic medicine?

| ○ Yes | ○ No | ○ Unsure |
| --- | --- | --- |

1. Have you personally experienced discriminatory behavior in academic medicine?

| ○ Yes | ○ No | ○ Unsure |
| --- | --- | --- |

1. Where do you go to seek supportive resources on campus when you witness discriminatory behavior?
2. Where do you go to seek supportive resources on campus when you personally experience discriminatory behavior?
3. About how often do you think about discriminatory behavior as it relates to your work?

| ○ Daily | ○ Weekly | ○ Monthly | ○ Every once in a  while | ○ Never |
| --- | --- | --- | --- | --- |

1. Over the last two years, have you completed any formal training related to the following topics?

|  | Yes | No | Unsure |
| --- | --- | --- | --- |
| Implicit or unconscious bias | ○ | ○ | ○ |
| Cultural competence/humility (e.g. Culture and Conversations) | ○ | ○ | ○ |
| Difficult, Crucial, or Courageous conversations (e.g. IU talks) | ○ | ○ | ○ |

1. If you completed any other related formal training, please list the topic(s) below:

Thank you for your responses!

**Responding to Instances of Racism, Discrimination, and Microaggressions**

**Evaluation and Postsurvey**

*Please take about* ***5 minutes*** *to complete this evaluation and postsurvey. This information will be used to evaluate the effectiveness of this workshop and improve for the future.*

1. Currently, how **comfortable** do you feel discussing the following topics with colleagues?

|  | Very comfortable | Somewhat comfortable | Somewhat uncomfortable | Very uncomfortable |
| --- | --- | --- | --- | --- |
| Race or ethnicity | ○ | ○ | ○ | ○ |
| Gender, gender identity, or gender expression | ○ | ○ | ○ | ○ |
| Sexual orientation | ○ | ○ | ○ | ○ |
| Spirituality and faith | ○ | ○ | ○ | ○ |

1. Currently, how **comfortable** do you feel discussing the following topics with trainees?

|  | Very comfortable | Somewhat comfortable | Somewhat uncomfortable | Very uncomfortable |
| --- | --- | --- | --- | --- |
| Race or ethnicity | ○ | ○ | ○ | ○ |
| Gender, gender identity, or gender expression | ○ | ○ | ○ | ○ |
| Sexual orientation | ○ | ○ | ○ | ○ |
| Spirituality and faith | ○ | ○ | ○ | ○ |

1. Currently, how **confident** do you feel initiating conversations about the following topics with colleagues?

|  | Very confident | Somewhat confident | Somewhat not confident | Not confident at all |
| --- | --- | --- | --- | --- |
| Race or ethnicity | ○ | ○ | ○ | ○ |
| Gender, gender identity, or gender expression | ○ | ○ | ○ | ○ |
| Sexual orientation | ○ | ○ | ○ | ○ |
| Spirituality and faith | ○ | ○ | ○ | ○ |

1. Currently, how **confident** do you feel initiating conversations about the following topics with trainees?

|  | Very confident | Somewhat confident | Somewhat not confident | Not confident at all |
| --- | --- | --- | --- | --- |
| Race or ethnicity | ○ | ○ | ○ | ○ |
| Gender, gender identity, or gender expression | ○ | ○ | ○ | ○ |
| Sexual orientation | ○ | ○ | ○ | ○ |
| Spirituality and faith | ○ | ○ | ○ | ○ |

**TURN OVER 🡪**

1. Please state your level of agreement with the following statements:

|  | Strongly agree | Agree | Undecided | Disagree | Strongly disagree | N/A |
| --- | --- | --- | --- | --- | --- | --- |
| The information presented in this workshop was useful to my professional work. | ○ | ○ | ○ | ○ | ○ | ○ |
| As a result of attending this workshop, I am better prepared to turn my current work into scholarship. | ○ | ○ | ○ | ○ | ○ | ○ |
| My professional work will improve as a result of attending this workshop. | ○ | ○ | ○ | ○ | ○ | ○ |
| I would recommend this workshop to a colleague. | ○ | ○ | ○ | ○ | ○ | ○ |

1. Prior to this workshop, how familiar were you with the material presented?

| **Previously very familiar**  5 | 4 | 3 | 2 | **Previously very unfamiliar**  1 |
| --- | --- | --- | --- | --- |
| ○ | ○ | ○ | ○ | ○ |

1. How much new information did you learn from this workshop?

| **Learned a great deal of information**  5 | 4 | 3 | 2 | **Did not learn anything new**  1 |
| --- | --- | --- | --- | --- |
| ○ | ○ | ○ | ○ | ○ |

1. What will you incorporate from this workshop into your work?
2. What part of the workshop resonated for you? How so?
3. What would you have liked to hear more about?
4. What suggestions do you have to improve this workshop so it would be more beneficial to your professional development?
