# Supplementary material for: “I Didn't Know What to Say”: Responding to Racism, Discrimination, and Microaggressions With the OWTFD Approach
Source: MedEdPORTAL. 2020 Jul 31;16:10971. doi: 10.15766/mep_2374-8265.10971 (PMC7394349; doi:10.15766/mep_2374-8265.10971)
Supplement: Supplementary file 1 — Workshop Agenda.docxPre- and Postsurvey.docxI Didn't Know What to Say.pptxSupplemental References.docxScenario Reenactment Script.docxScenario Guest Reflections.docxReflection Exercise.docx [file mep_2374-8265.10971-s001.zip › C. I Didn't Know What to Say.pptx]

## Slide 1
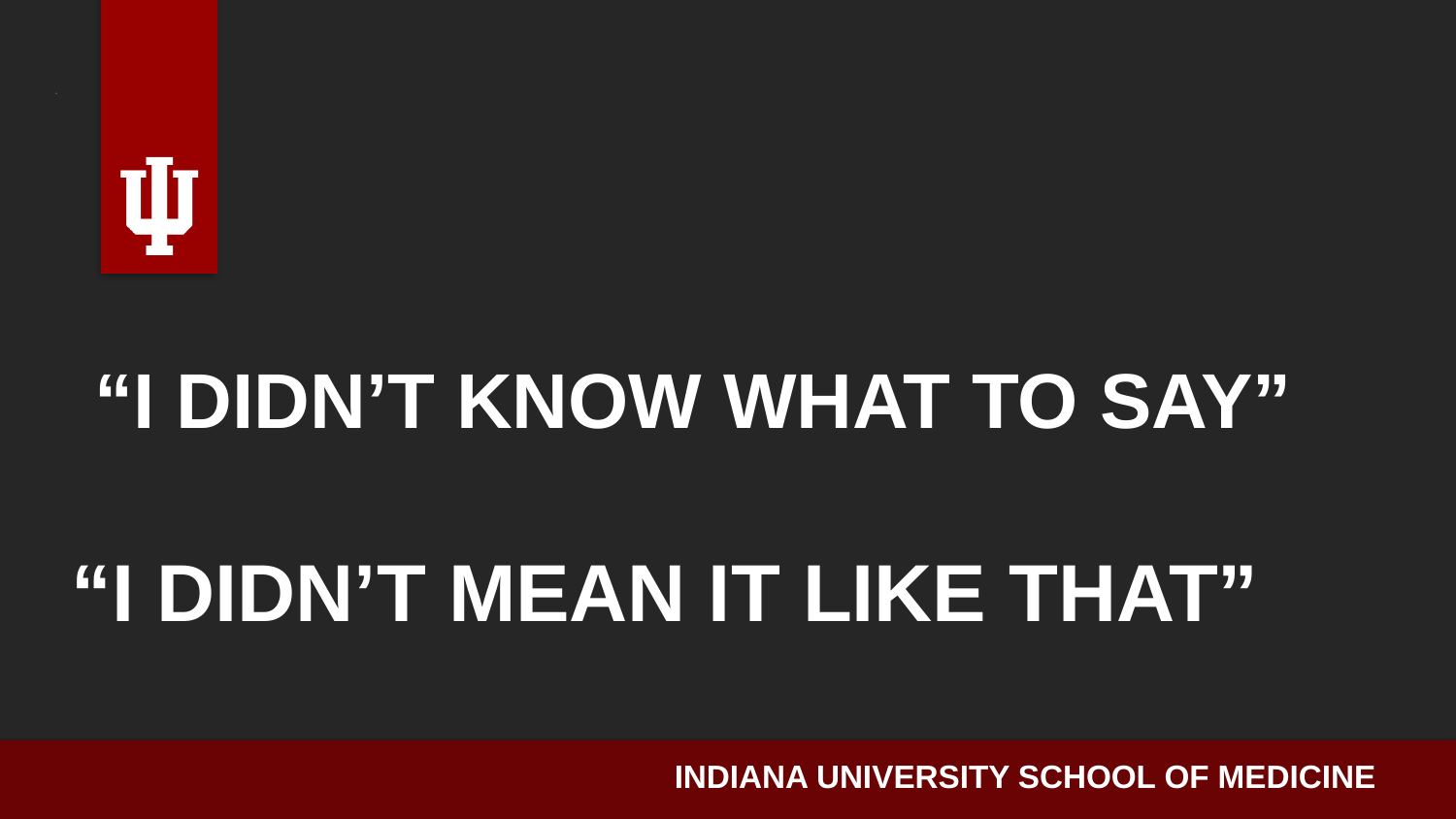

# “I DIDN’T KNOW WHAT TO SAY”
“I DIDN’T MEAN IT LIKE THAT”

## Slide 2
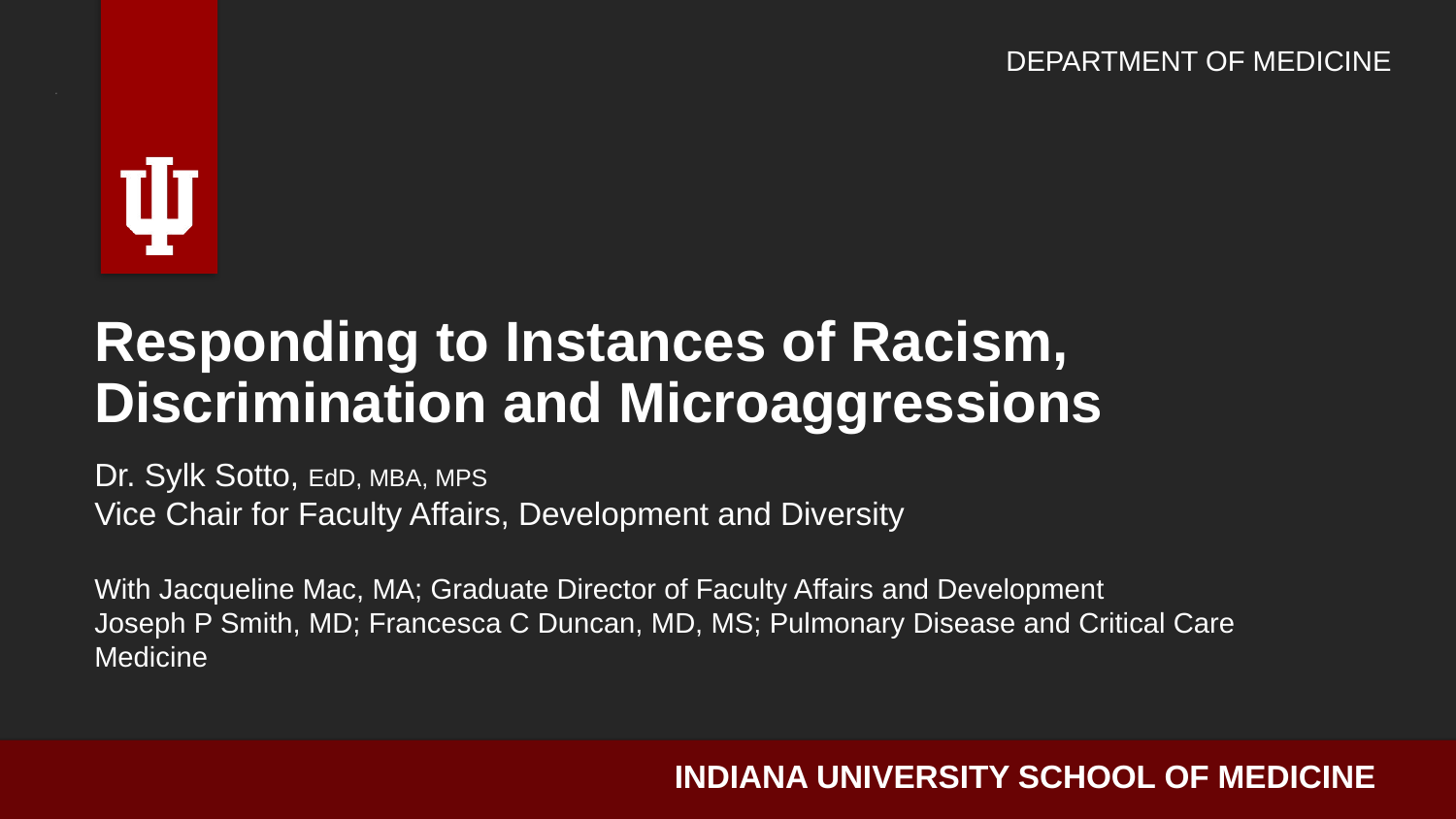

DEPARTMENT OF MEDICINE
# Responding to Instances of Racism, Discrimination and Microaggressions
Dr. Sylk Sotto, EdD, MBA, MPS
Vice Chair for Faculty Affairs, Development and Diversity
With Jacqueline Mac, MA; Graduate Director of Faculty Affairs and Development
Joseph P Smith, MD; Francesca C Duncan, MD, MS; Pulmonary Disease and Critical Care Medicine

## Slide 3
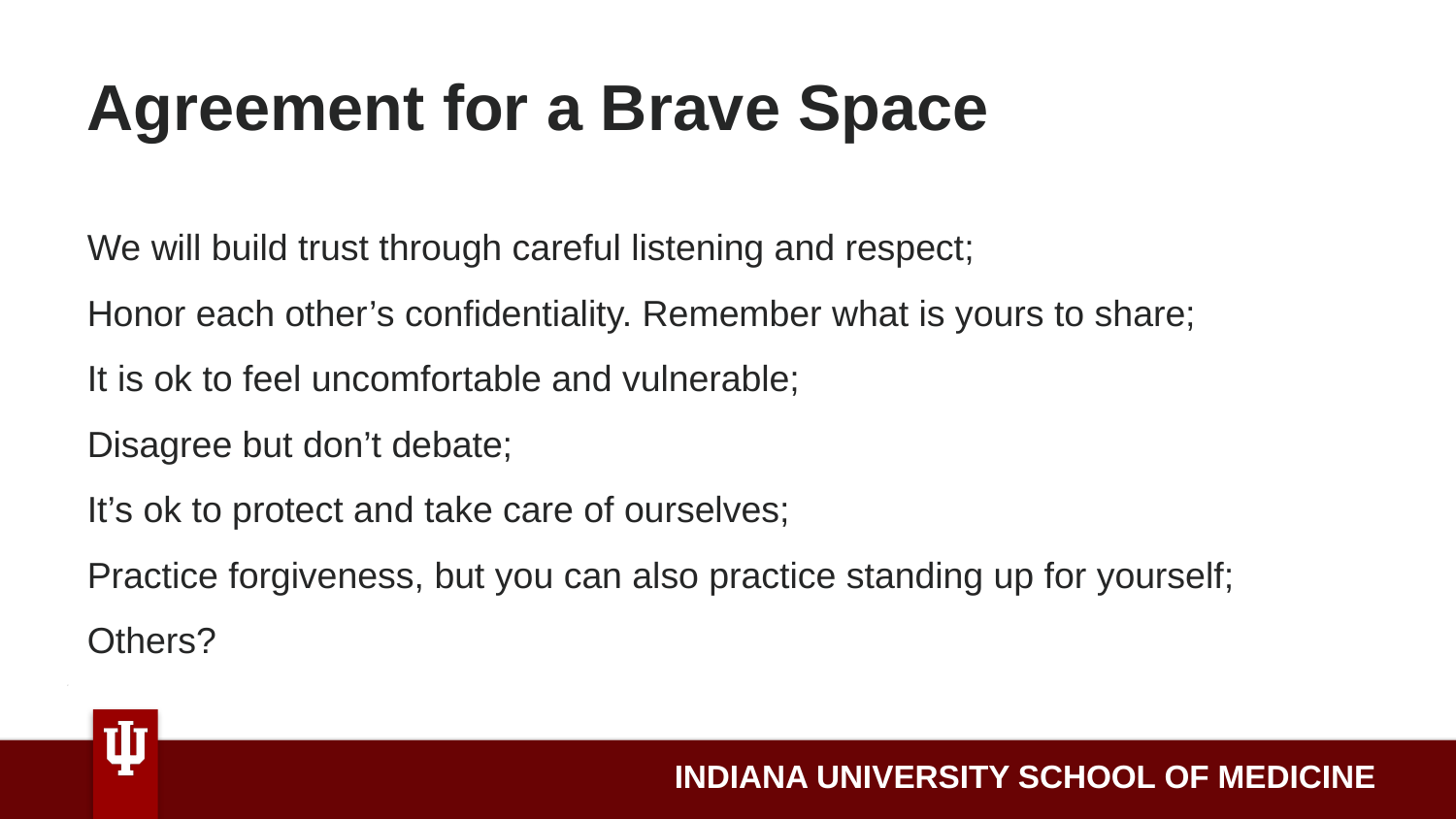

Agreement for a Brave Space
We will build trust through careful listening and respect;
Honor each other’s confidentiality. Remember what is yours to share;
It is ok to feel uncomfortable and vulnerable;
Disagree but don’t debate;
It’s ok to protect and take care of ourselves;
Practice forgiveness, but you can also practice standing up for yourself;
Others?

## Slide 4
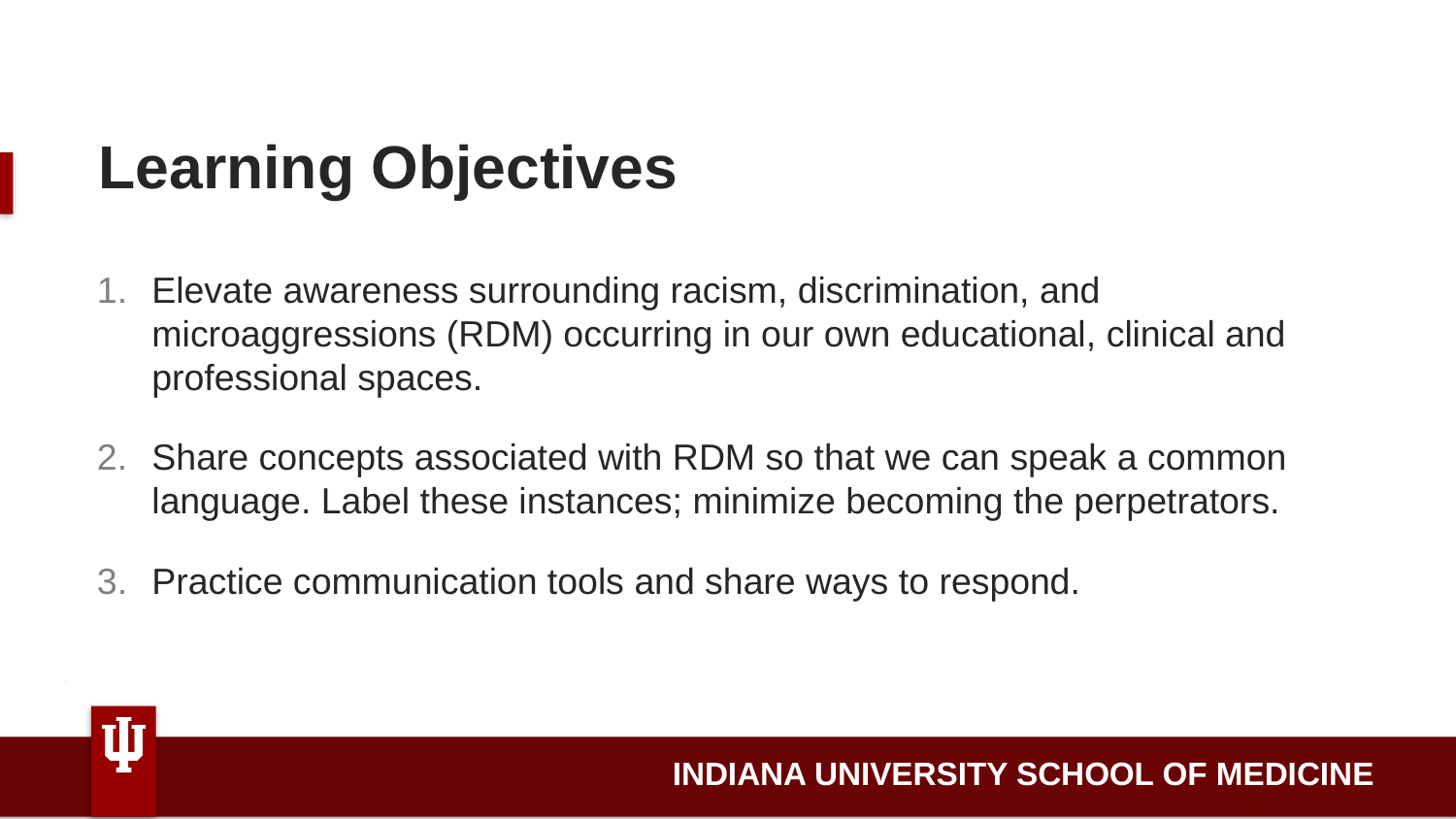

# Learning Objectives
Elevate awareness surrounding racism, discrimination, and microaggressions (RDM) occurring in our own educational, clinical and professional spaces.
Share concepts associated with RDM so that we can speak a common language. Label these instances; minimize becoming the perpetrators.
Practice communication tools and share ways to respond.

## Slide 5
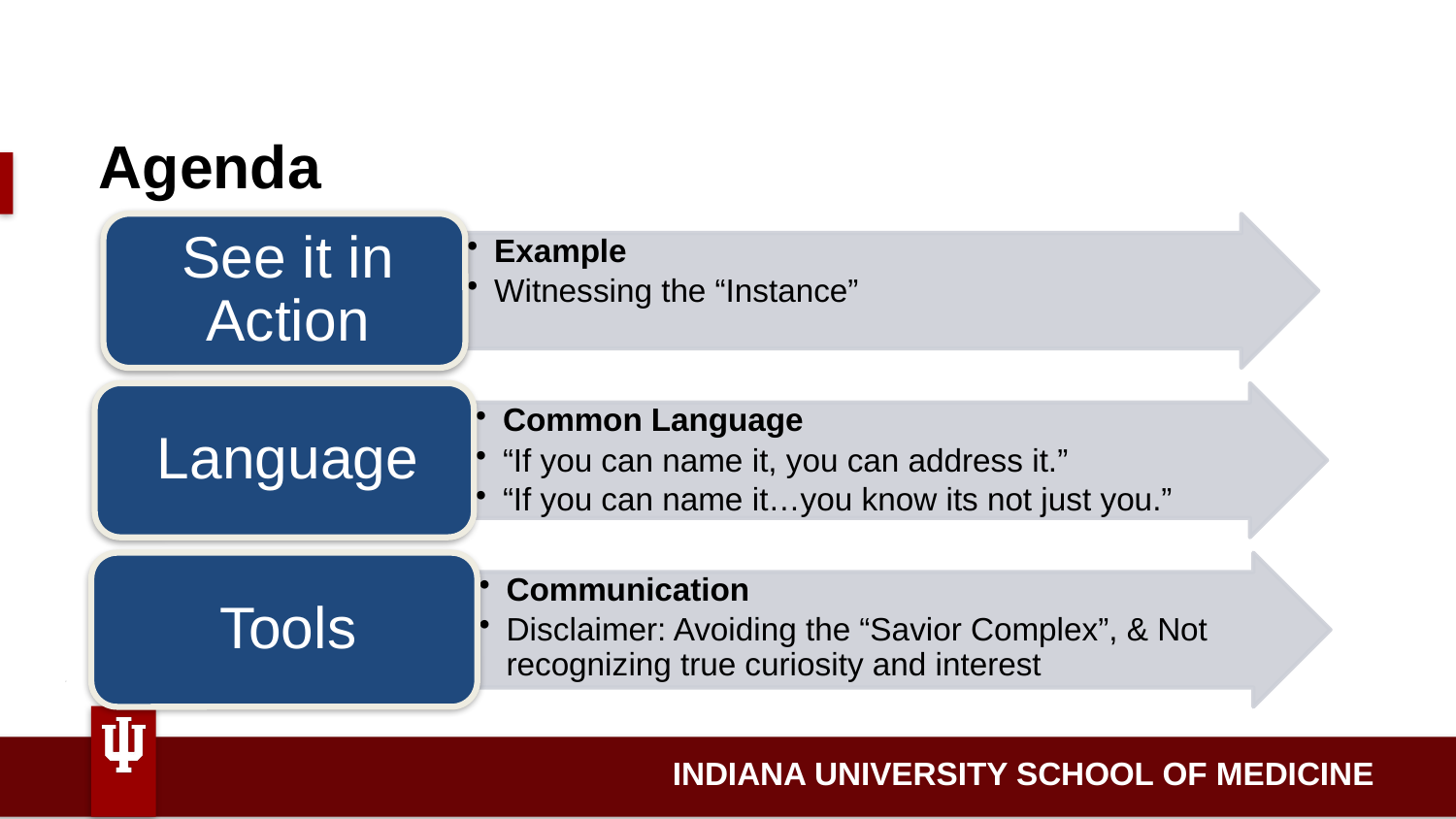

# Agenda

## Slide 6
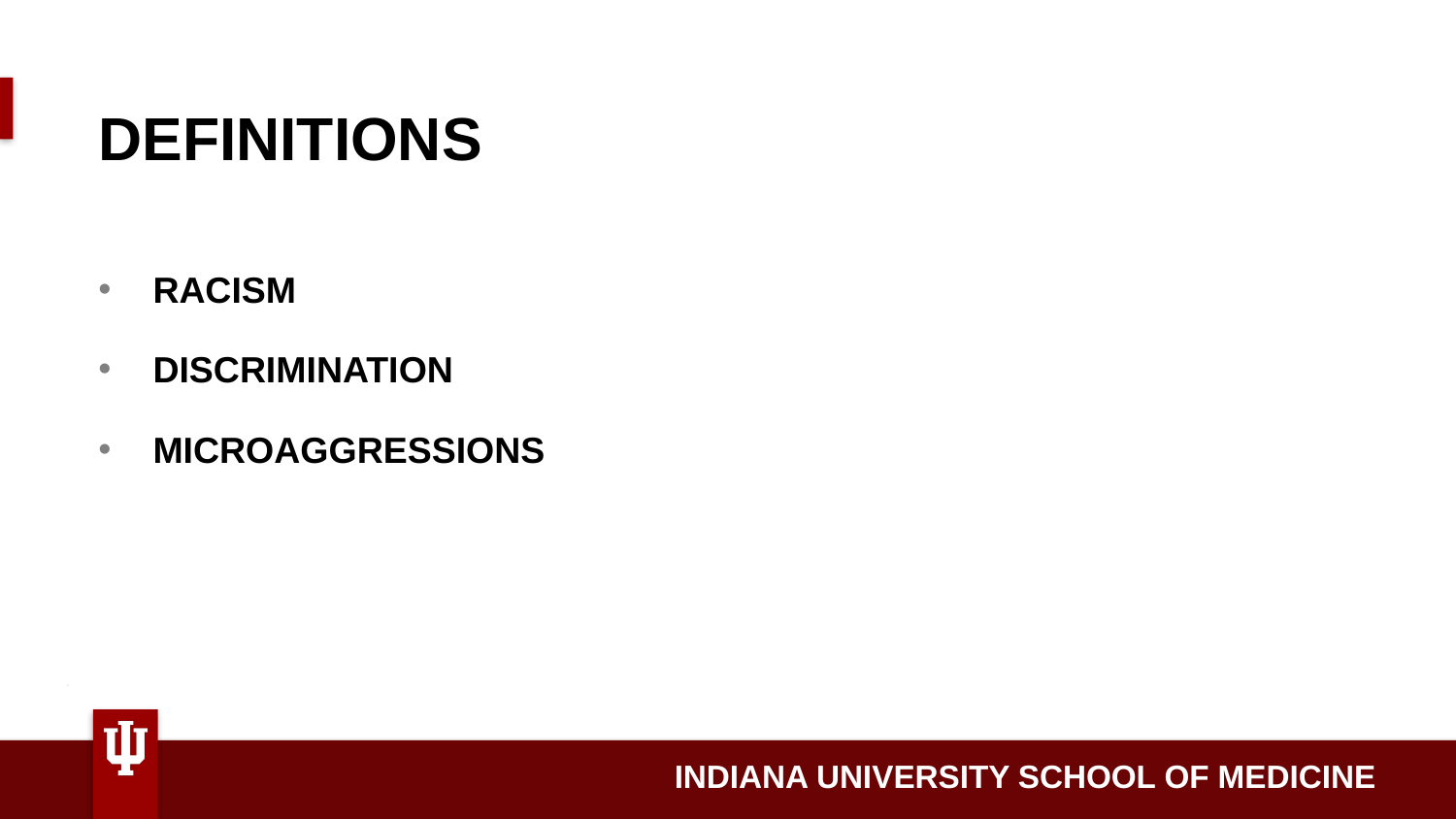

# DEFINITIONS
RACISM
DISCRIMINATION
MICROAGGRESSIONS

## Slide 7
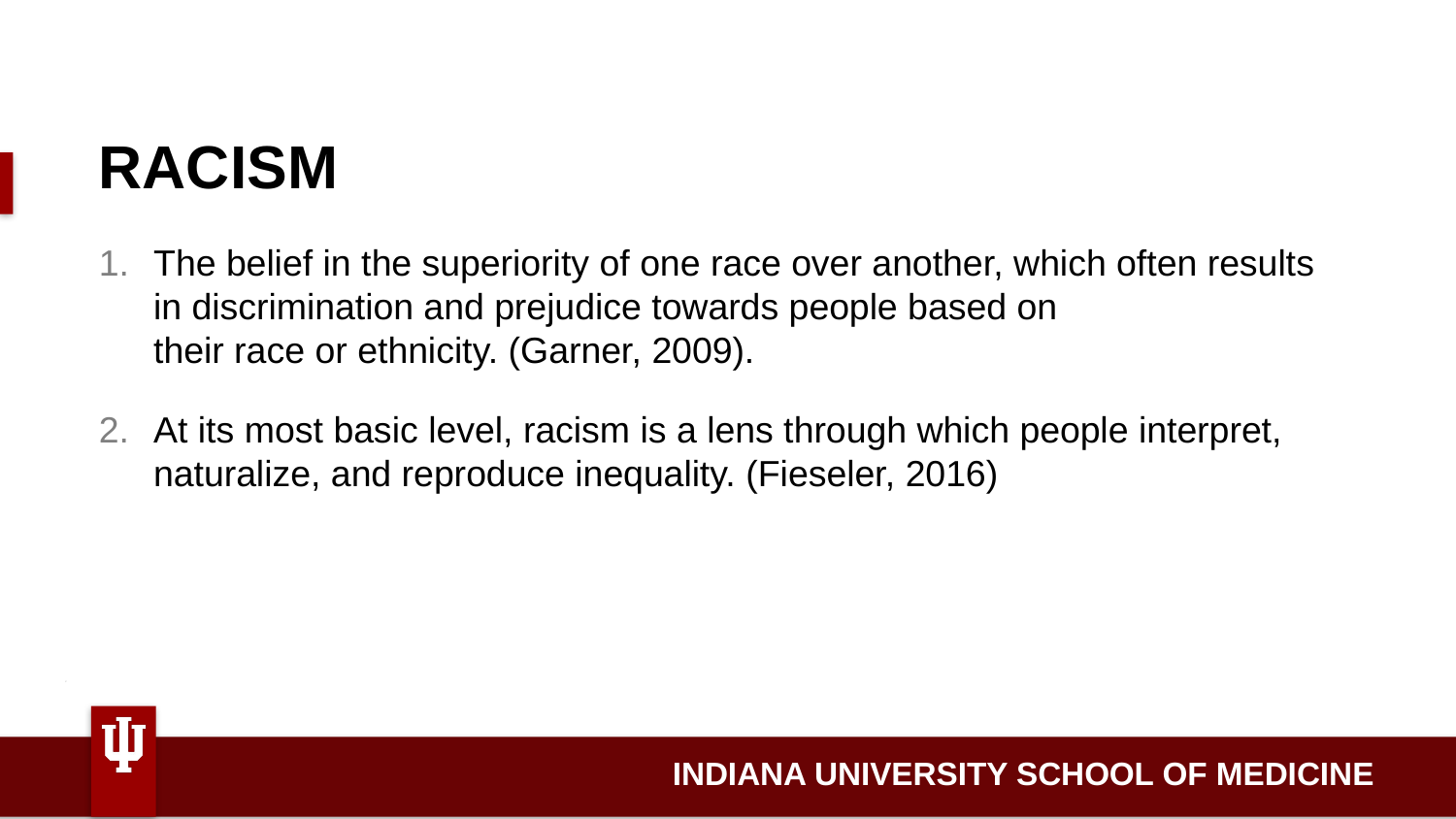

# RACISM
The belief in the superiority of one race over another, which often results in discrimination and prejudice towards people based on their race or ethnicity. (Garner, 2009).
At its most basic level, racism is a lens through which people interpret, naturalize, and reproduce inequality. (Fieseler, 2016)

## Slide 8
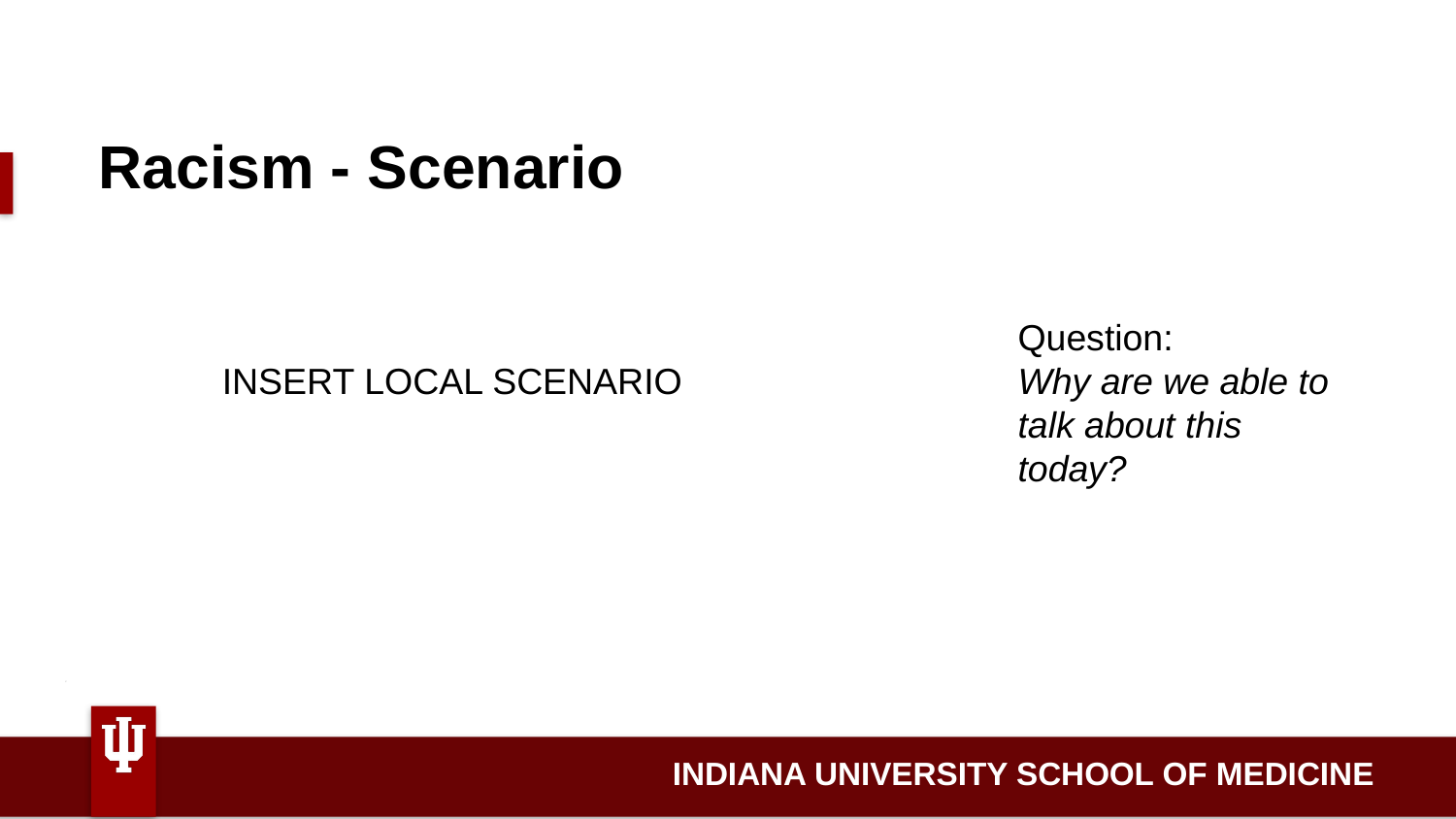

# Racism - Scenario
Question:
Why are we able to talk about this today?
INSERT LOCAL SCENARIO

## Slide 9
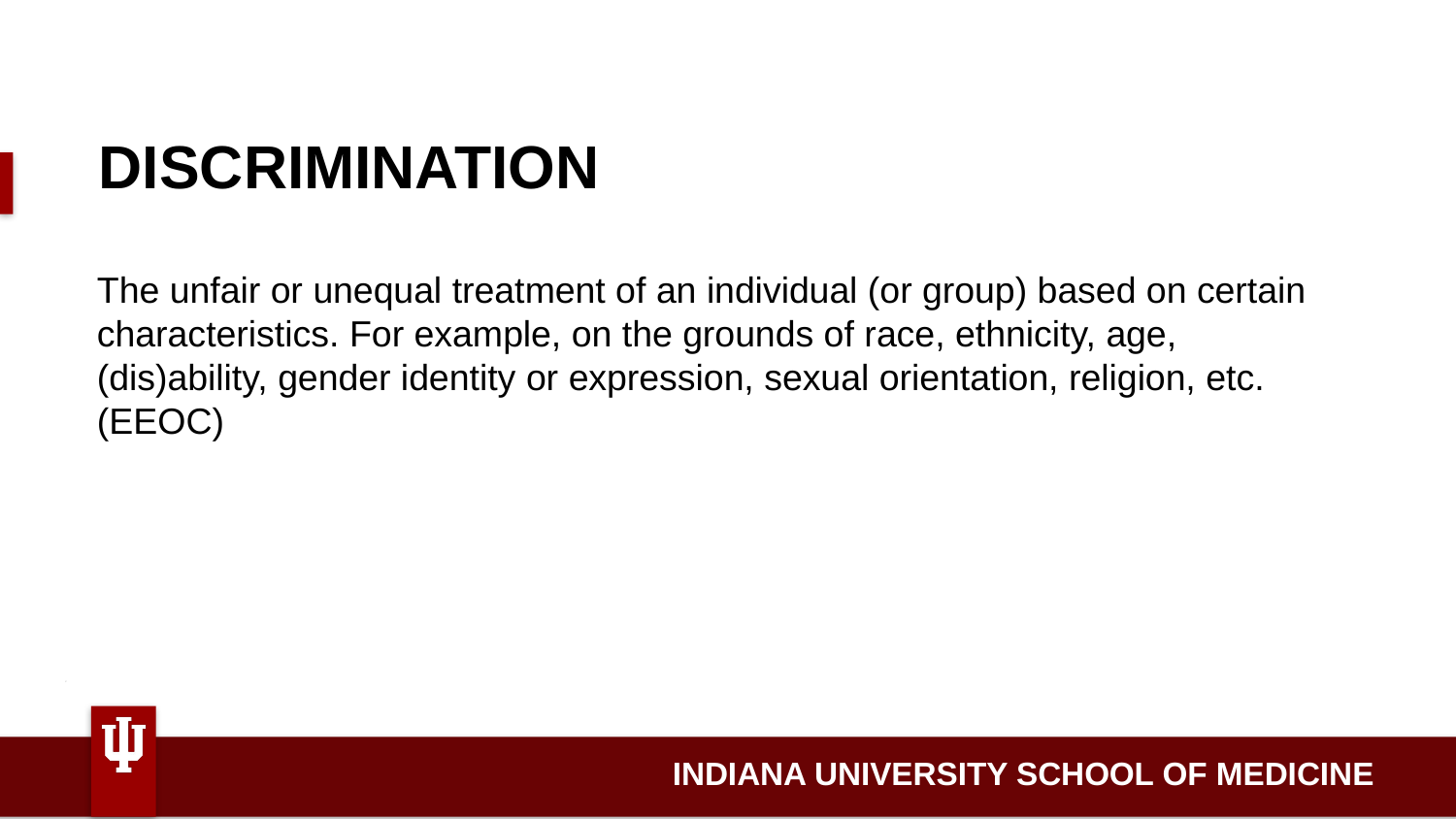

# DISCRIMINATION
The unfair or unequal treatment of an individual (or group) based on certain characteristics. For example, on the grounds of race, ethnicity, age, (dis)ability, gender identity or expression, sexual orientation, religion, etc. (EEOC)

## Slide 10
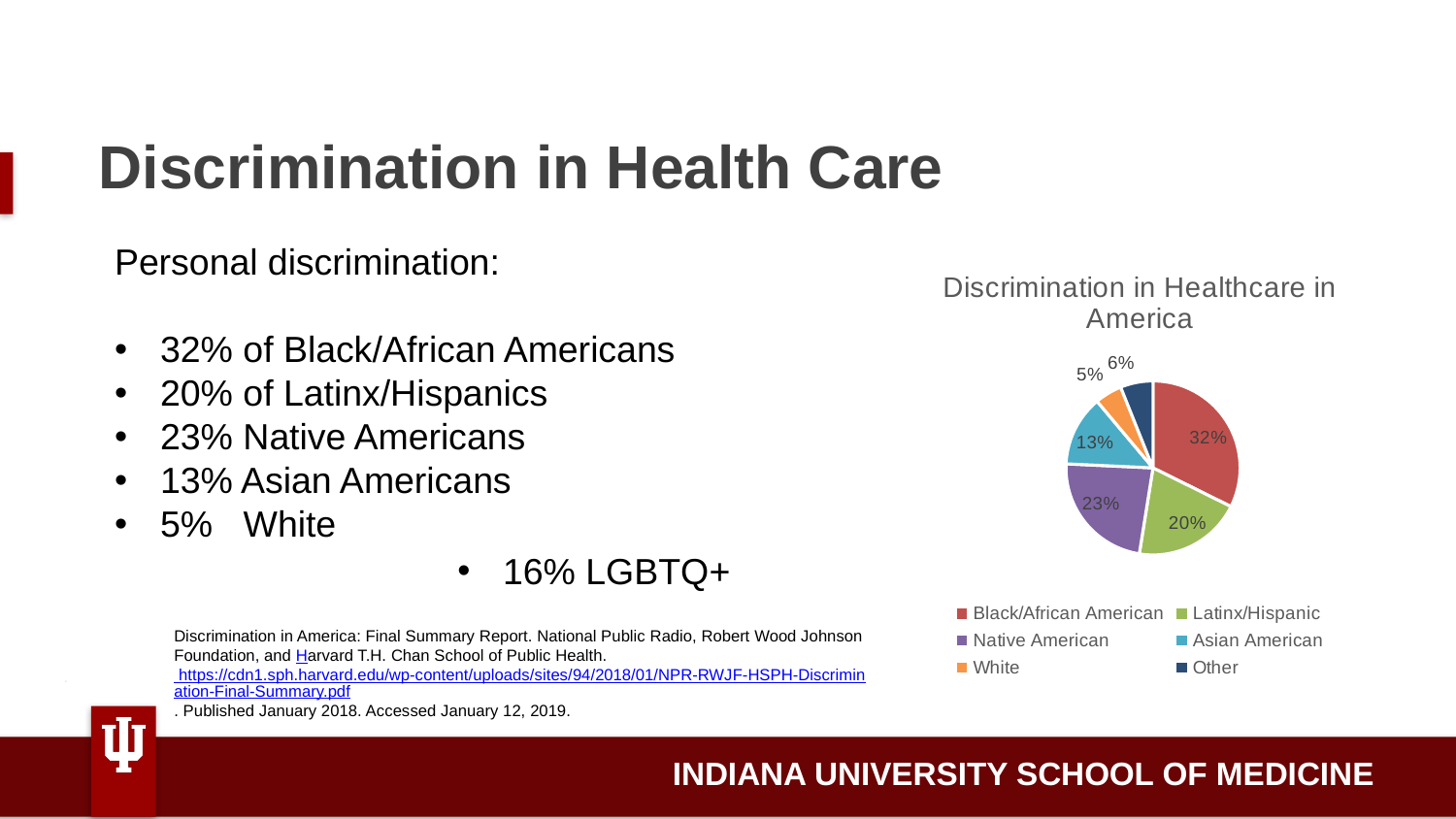

# Discrimination in Health Care
Personal discrimination:
32% of Black/African Americans
20% of Latinx/Hispanics
23% Native Americans
13% Asian Americans
5% White
### Chart: Discrimination in Healthcare in America
| Category | |
|---|---|
| Discrimination in Health Care in America | None |
| Black/African American | 0.32 |
| Latinx/Hispanic | 0.2 |
| Native American | 0.23 |
| Asian American | 0.13 |
| White | 0.05 |
| Other | 0.06 |16% LGBTQ+
Discrimination in America: Final Summary Report. National Public Radio, Robert Wood Johnson Foundation, and Harvard T.H. Chan School of Public Health. https://cdn1.sph.harvard.edu/wp-content/uploads/sites/94/2018/01/NPR-RWJF-HSPH-Discrimination-Final-Summary.pdf. Published January 2018. Accessed January 12, 2019.

## Slide 11
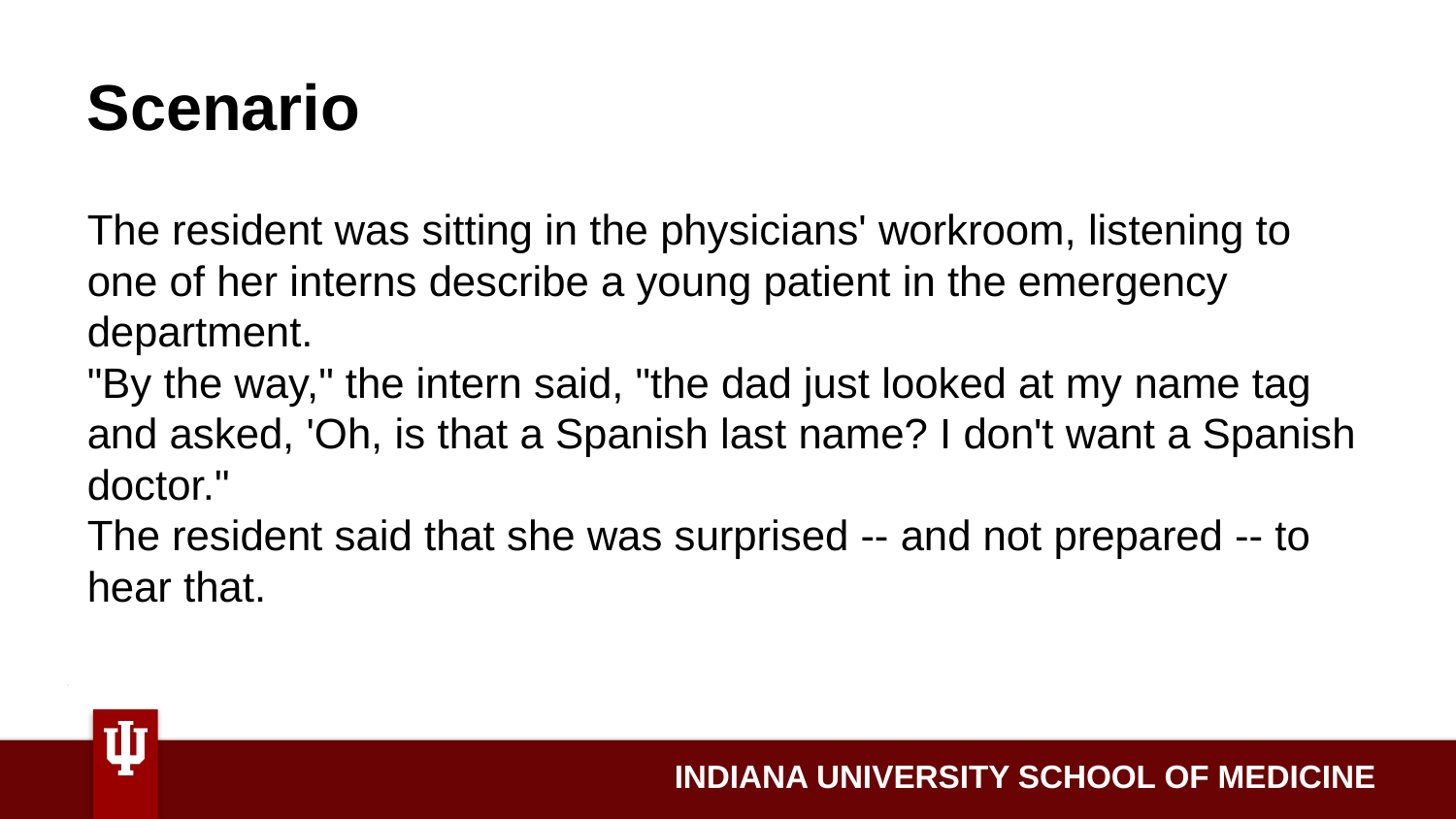

Scenario
The resident was sitting in the physicians' workroom, listening to one of her interns describe a young patient in the emergency department.
"By the way," the intern said, "the dad just looked at my name tag and asked, 'Oh, is that a Spanish last name? I don't want a Spanish doctor."
The resident said that she was surprised -- and not prepared -- to hear that.

## Slide 12
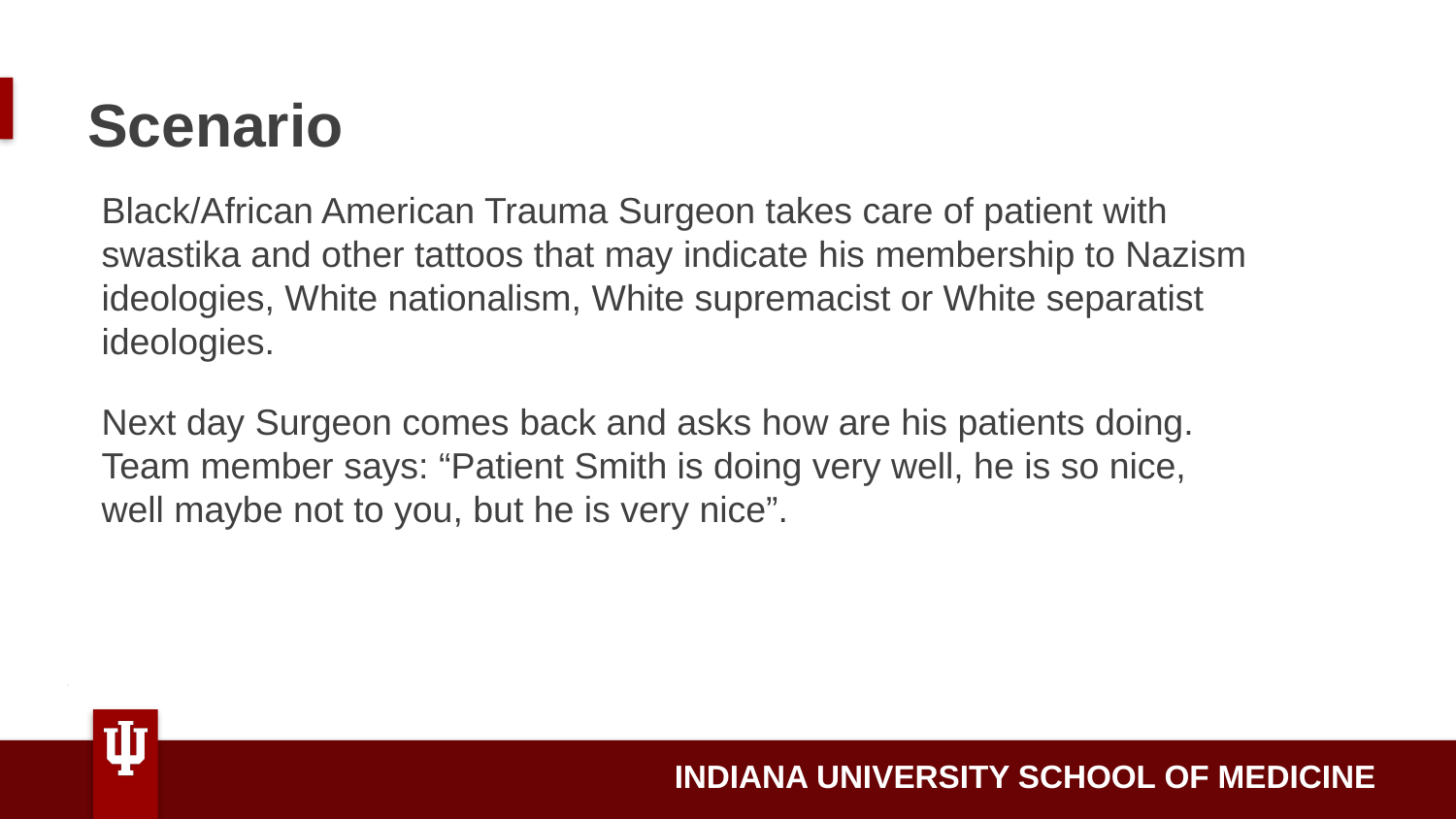

# Scenario
Black/African American Trauma Surgeon takes care of patient with swastika and other tattoos that may indicate his membership to Nazism ideologies, White nationalism, White supremacist or White separatist ideologies.
Next day Surgeon comes back and asks how are his patients doing. Team member says: “Patient Smith is doing very well, he is so nice, well maybe not to you, but he is very nice”.

## Slide 13
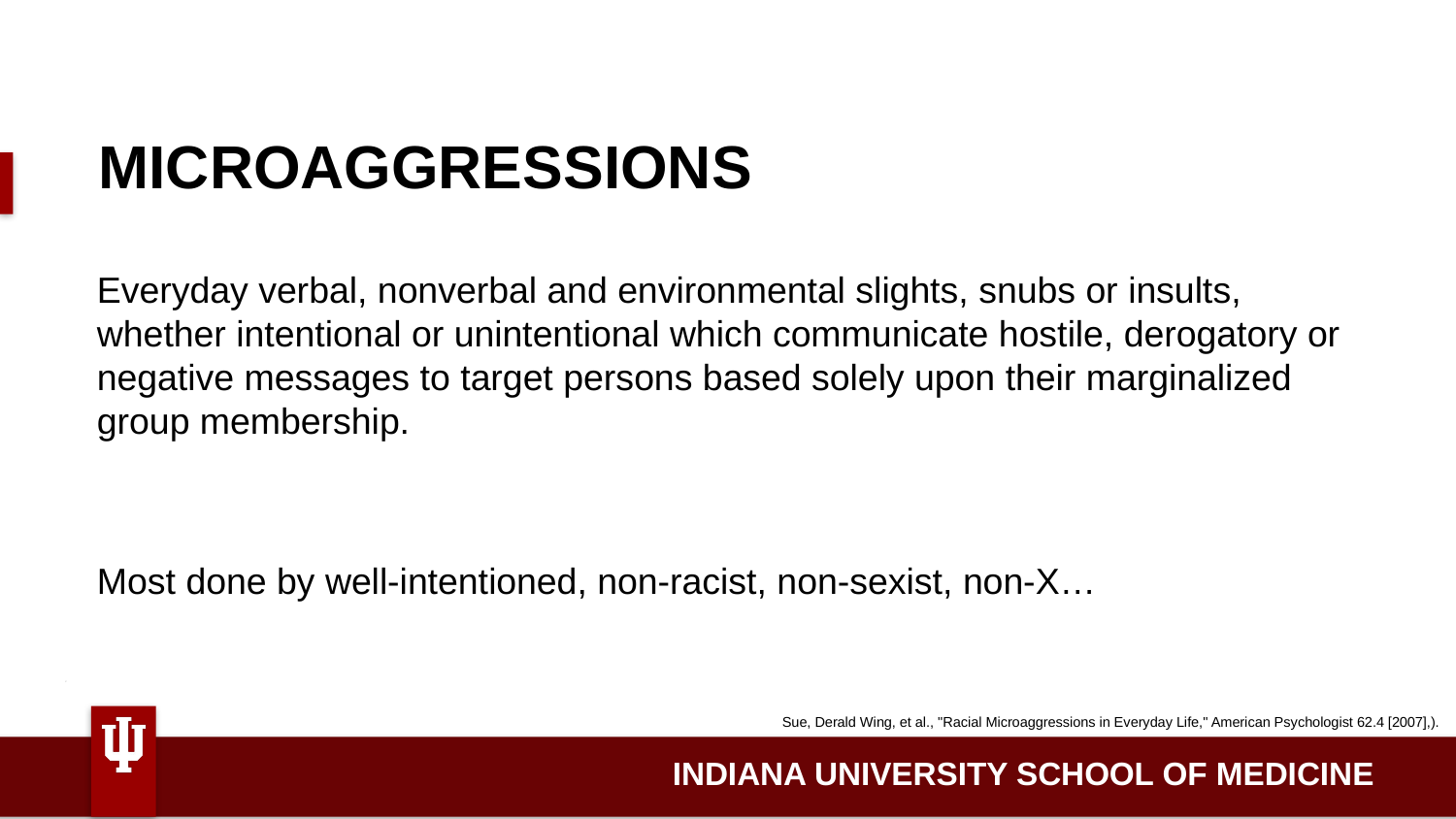

# MICROAGGRESSIONS
Everyday verbal, nonverbal and environmental slights, snubs or insults, whether intentional or unintentional which communicate hostile, derogatory or negative messages to target persons based solely upon their marginalized group membership.
Most done by well-intentioned, non-racist, non-sexist, non-X…
Sue, Derald Wing, et al., "Racial Microaggressions in Everyday Life," American Psychologist 62.4 [2007],).

## Slide 14
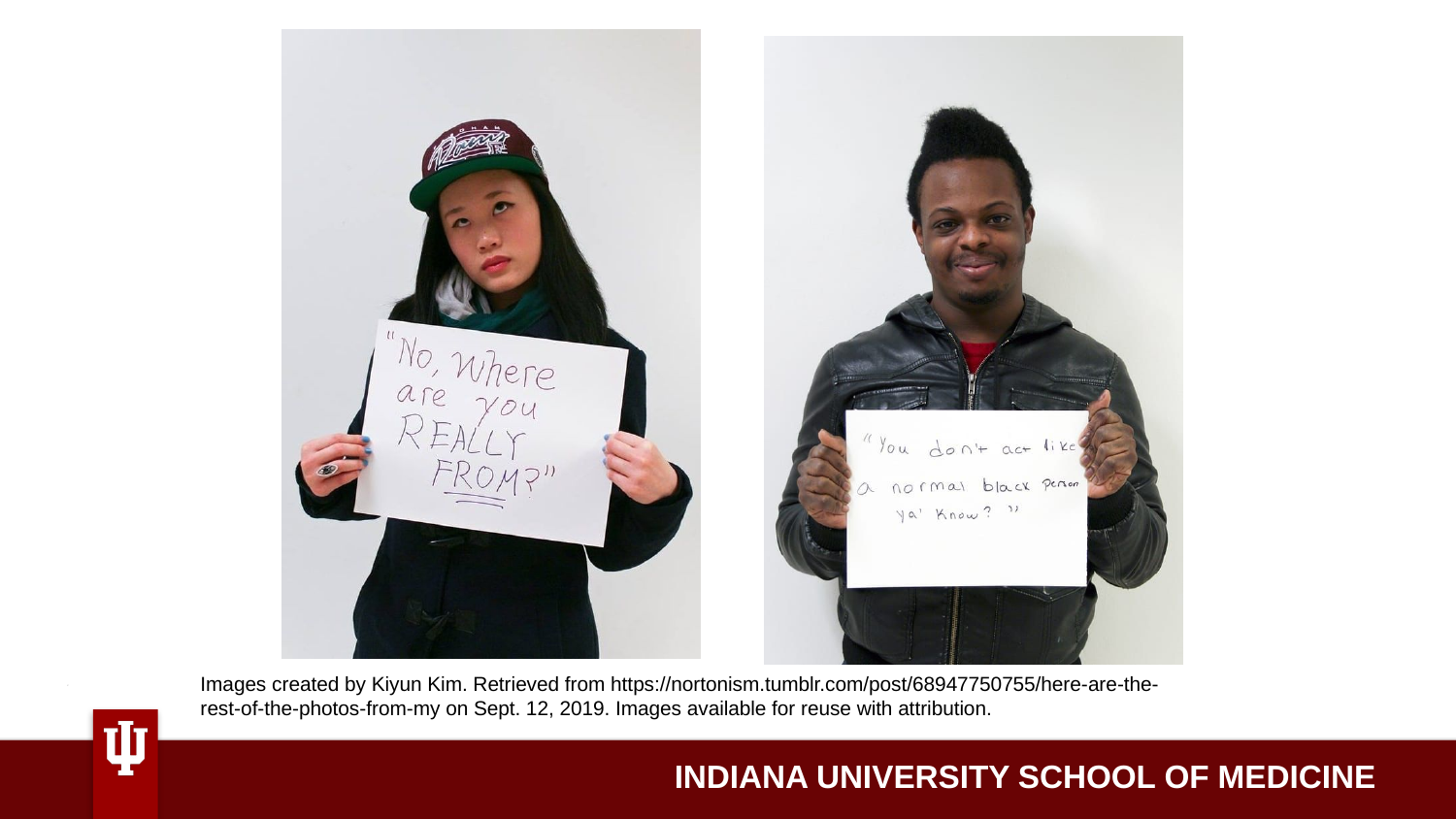

Images created by Kiyun Kim. Retrieved from https://nortonism.tumblr.com/post/68947750755/here-are-the-rest-of-the-photos-from-my on Sept. 12, 2019. Images available for reuse with attribution.

## Slide 15
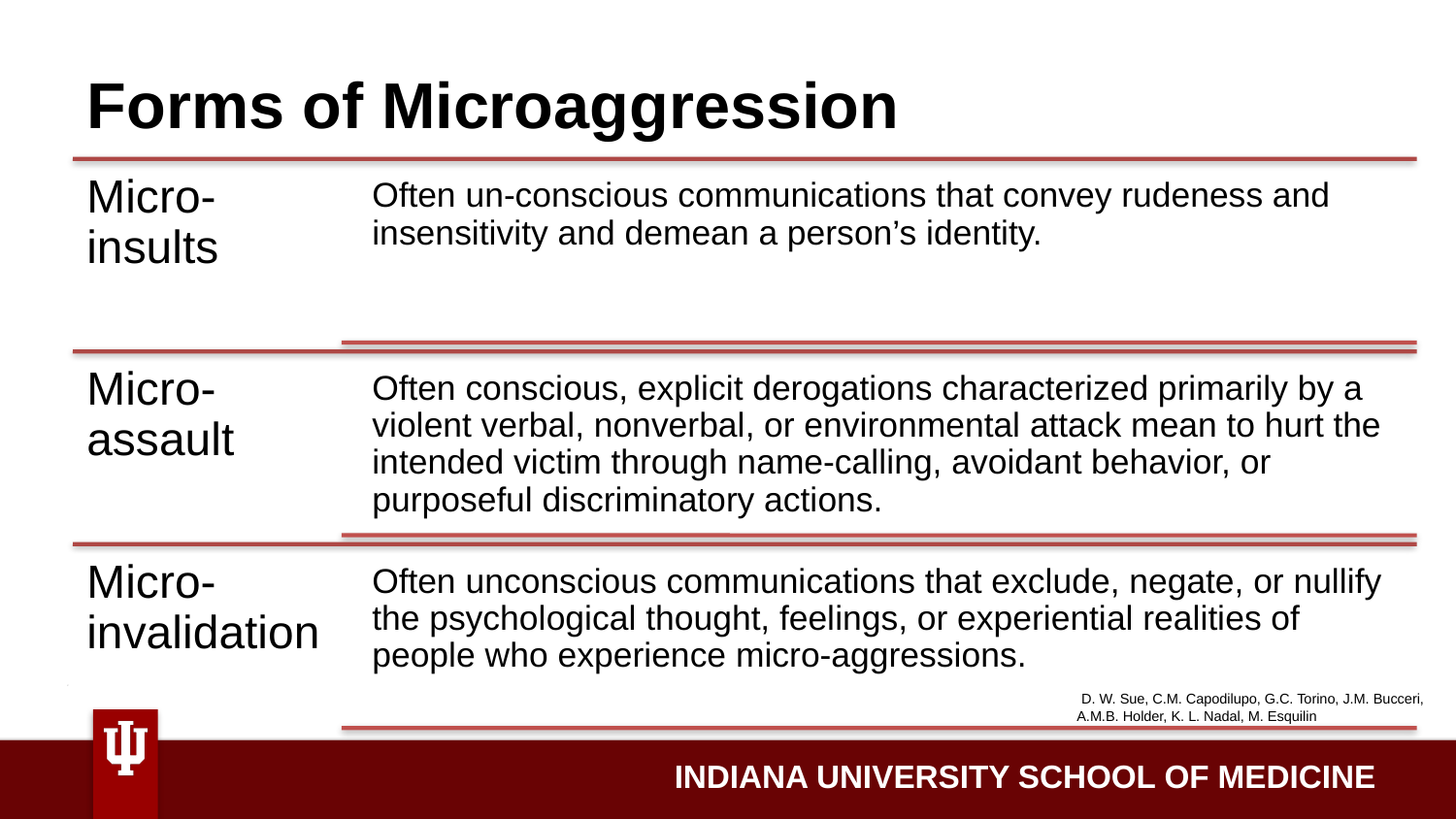

Forms of Microaggression
 D. W. Sue, C.M. Capodilupo, G.C. Torino, J.M. Bucceri,
A.M.B. Holder, K. L. Nadal, M. Esquilin

## Slide 16
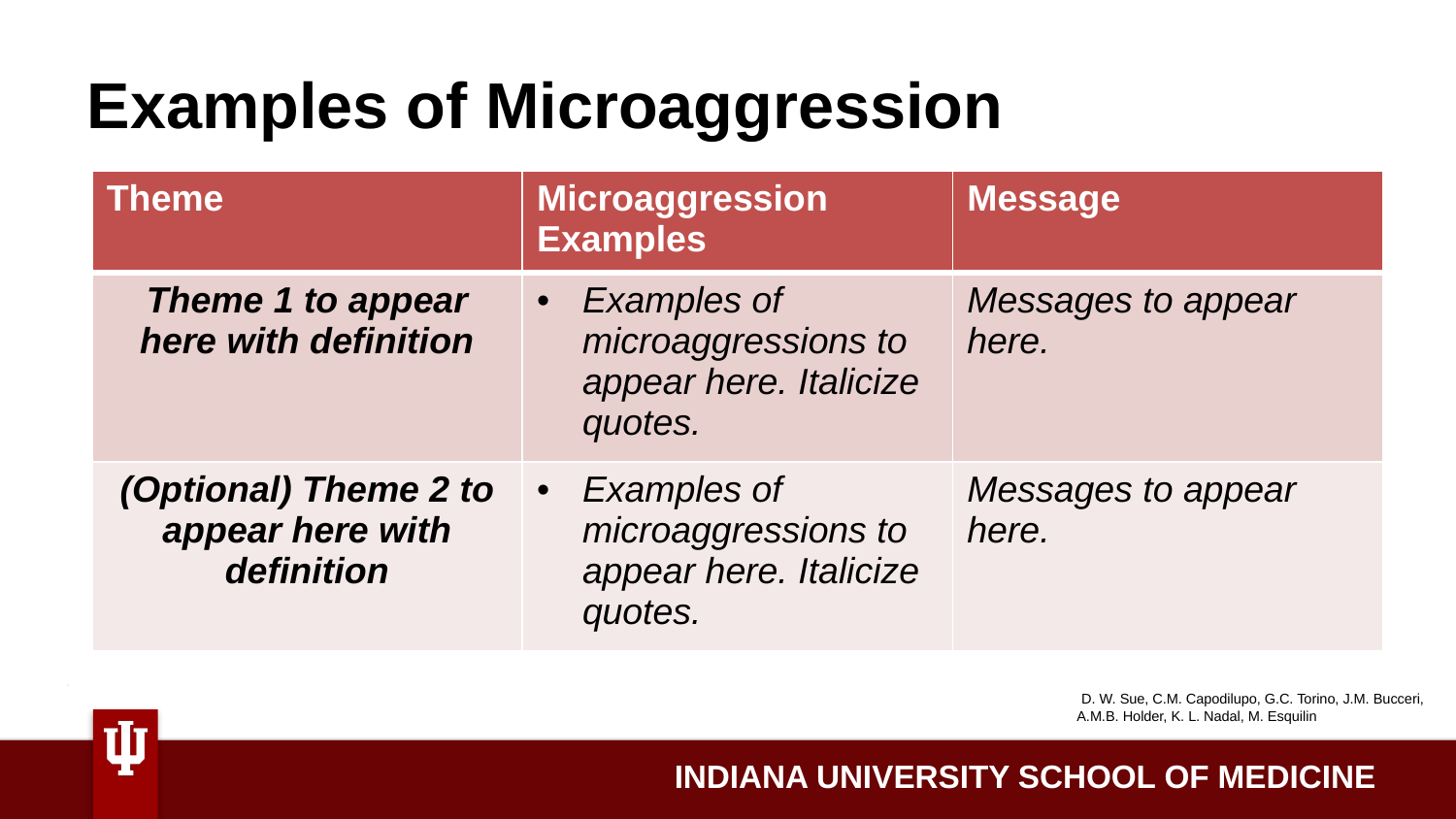

Examples of Microaggression
| Theme | Microaggression Examples | Message |
| --- | --- | --- |
| Theme 1 to appear here with definition | Examples of microaggressions to appear here. Italicize quotes. | Messages to appear here. |
| (Optional) Theme 2 to appear here with definition | Examples of microaggressions to appear here. Italicize quotes. | Messages to appear here. |
 D. W. Sue, C.M. Capodilupo, G.C. Torino, J.M. Bucceri,
A.M.B. Holder, K. L. Nadal, M. Esquilin

## Slide 17
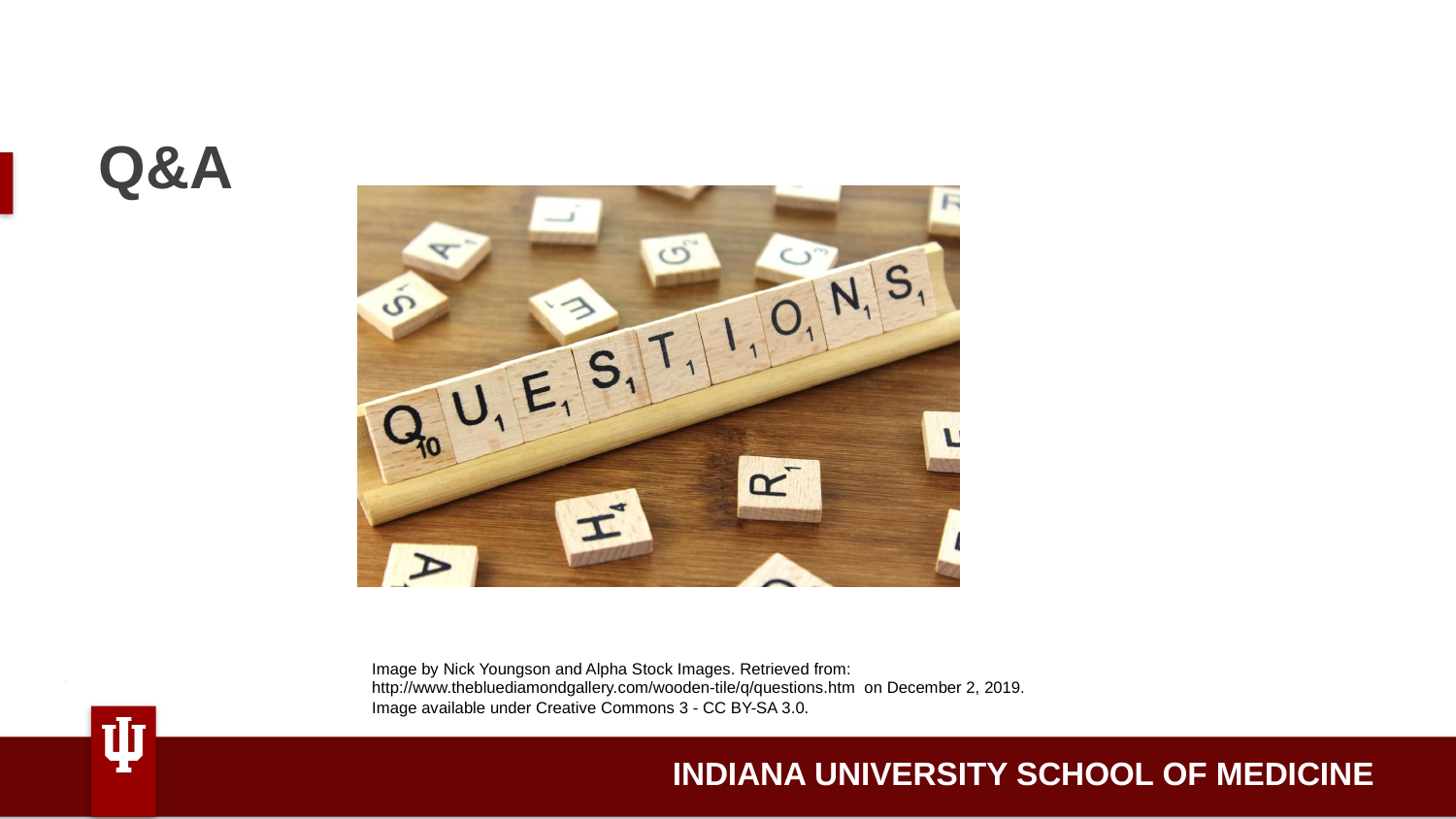

# Q&A
Image by Nick Youngson and Alpha Stock Images. Retrieved from: http://www.thebluediamondgallery.com/wooden-tile/q/questions.htm on December 2, 2019. Image available under Creative Commons 3 - CC BY-SA 3.0.
This Photo by Unknown Author is licensed under CC BY-SA

## Slide 18
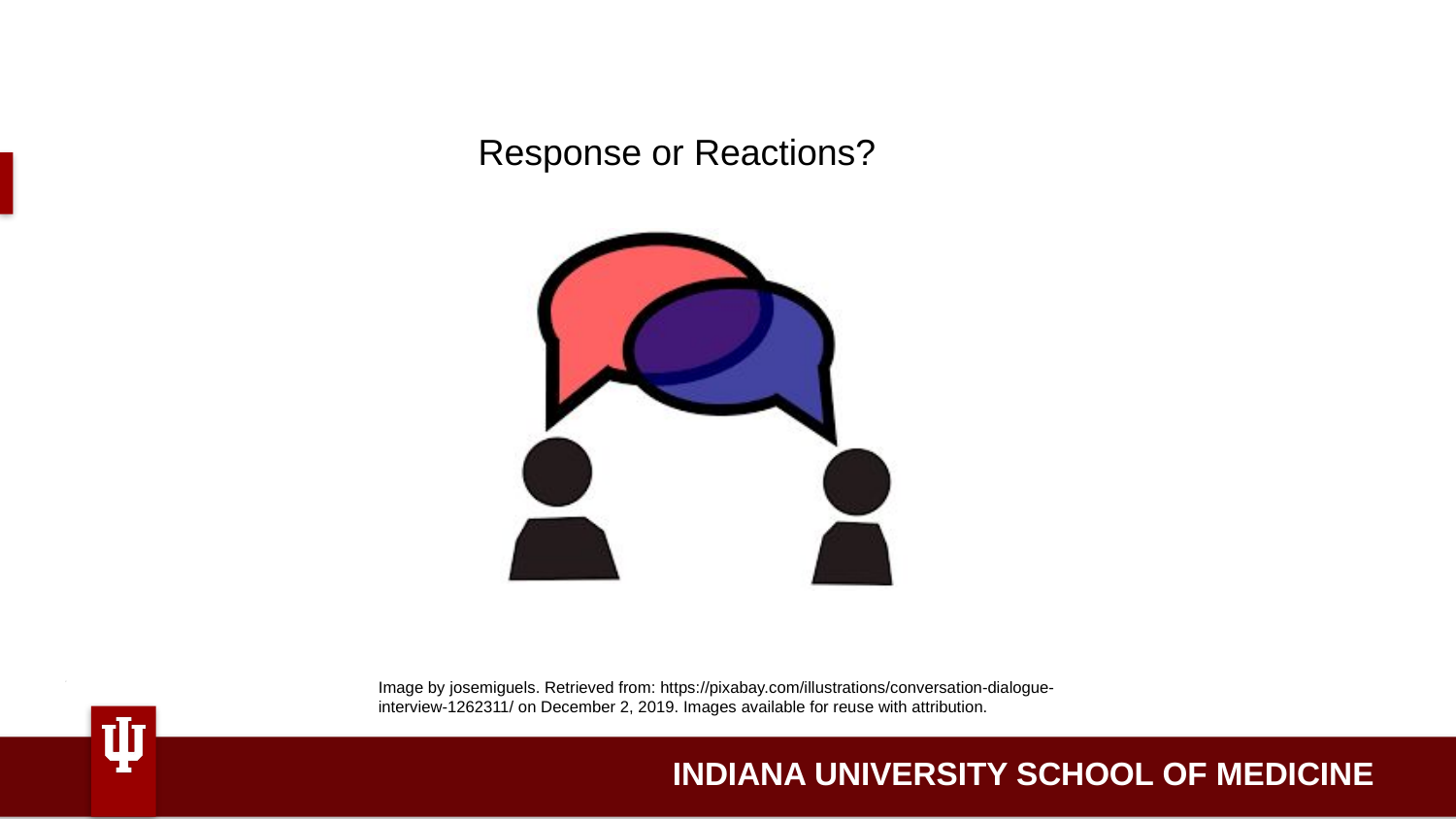

Response or Reactions?
Image by josemiguels. Retrieved from: https://pixabay.com/illustrations/conversation-dialogue-interview-1262311/ on December 2, 2019. Images available for reuse with attribution.

## Slide 19
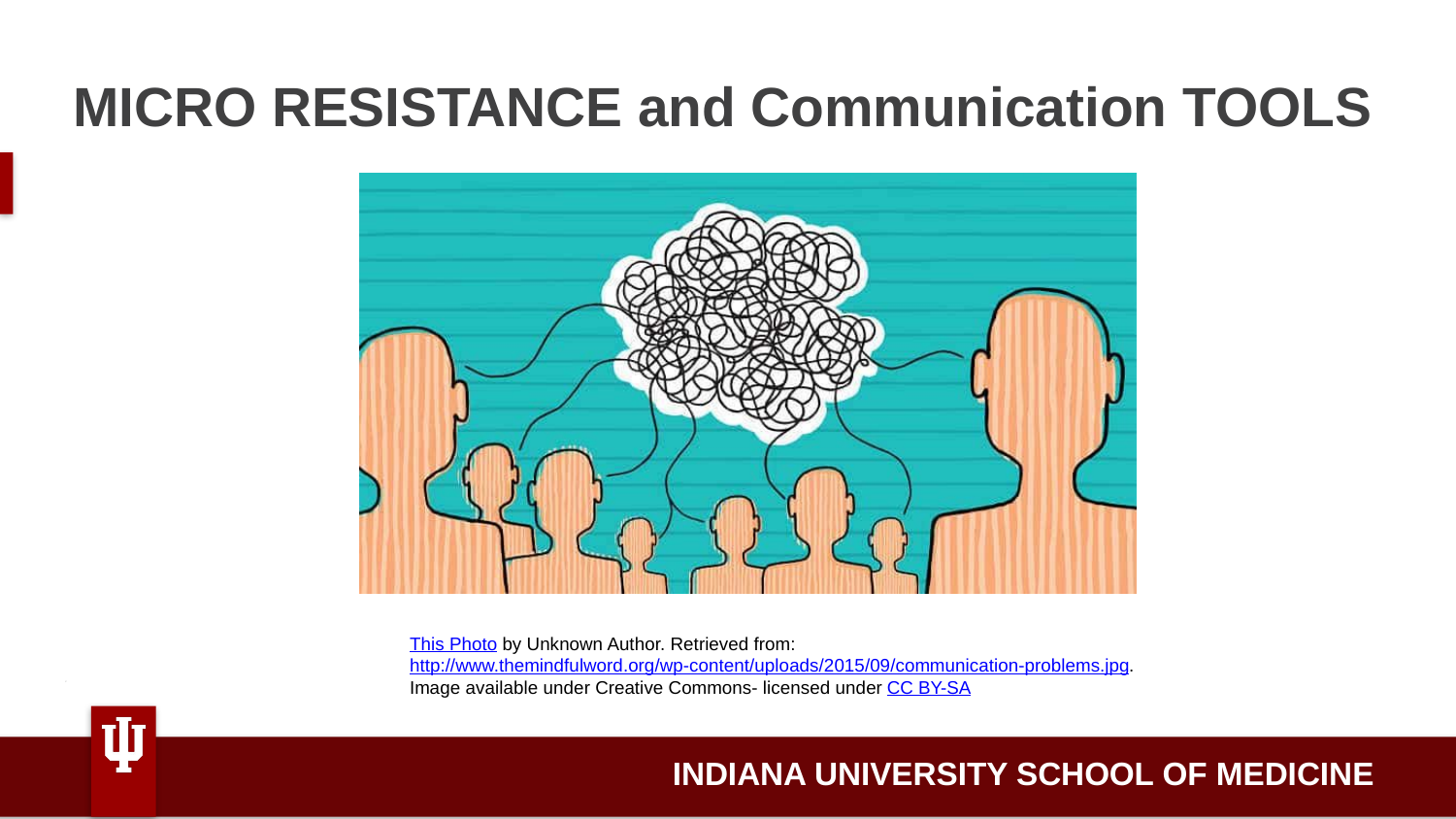

MICRO RESISTANCE and Communication TOOLS
This Photo by Unknown Author. Retrieved from: http://www.themindfulword.org/wp-content/uploads/2015/09/communication-problems.jpg. Image available under Creative Commons- licensed under CC BY-SA

## Slide 20
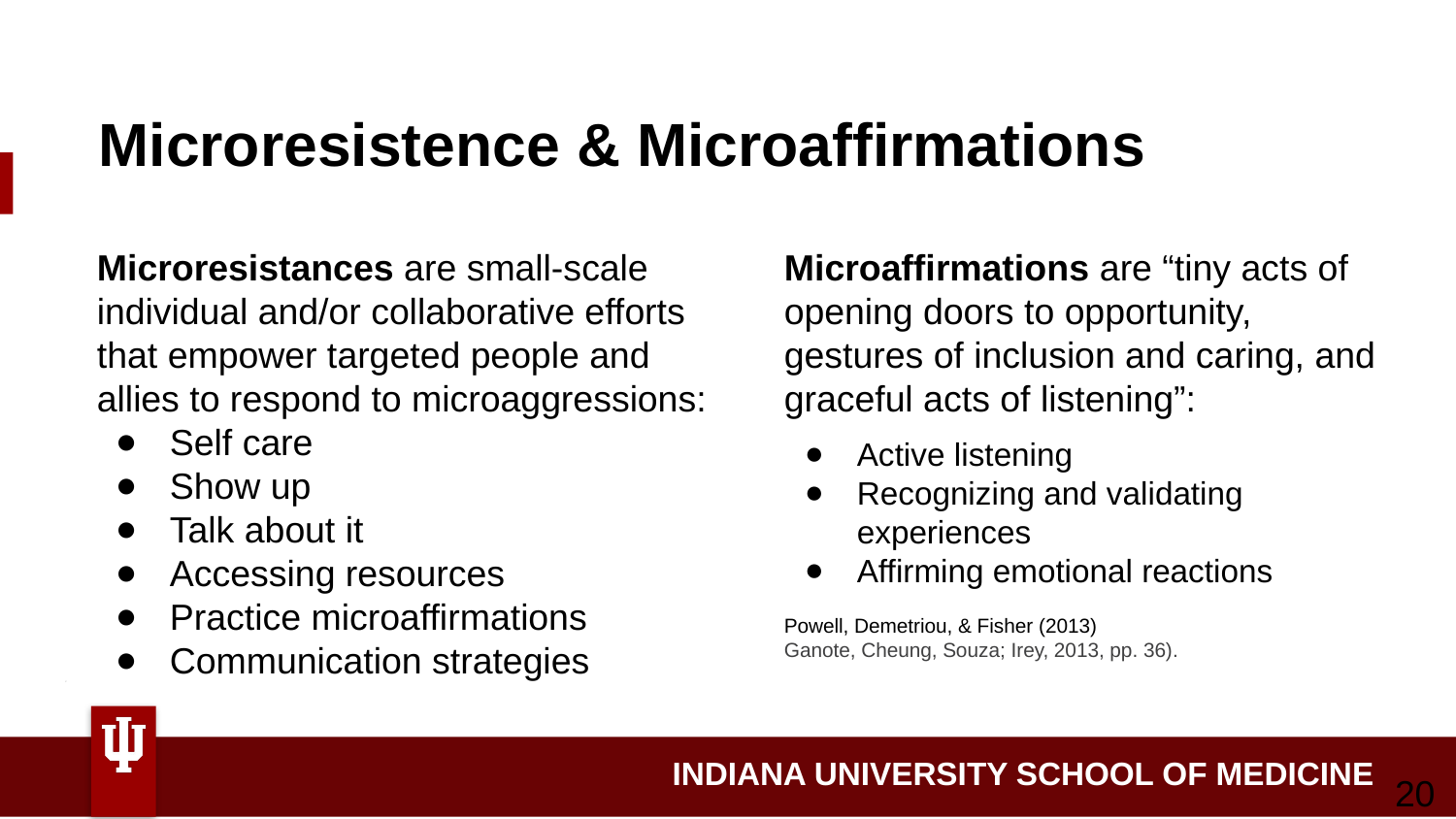

# Microresistence & Microaffirmations
Microresistances are small-scale individual and/or collaborative efforts that empower targeted people and allies to respond to microaggressions:
Self care
Show up
Talk about it
Accessing resources
Practice microaffirmations
Communication strategies
Microaffirmations are “tiny acts of opening doors to opportunity, gestures of inclusion and caring, and graceful acts of listening”:
Active listening
Recognizing and validating experiences
Affirming emotional reactions
Powell, Demetriou, & Fisher (2013)
Ganote, Cheung, Souza; Irey, 2013, pp. 36).
20

## Slide 21
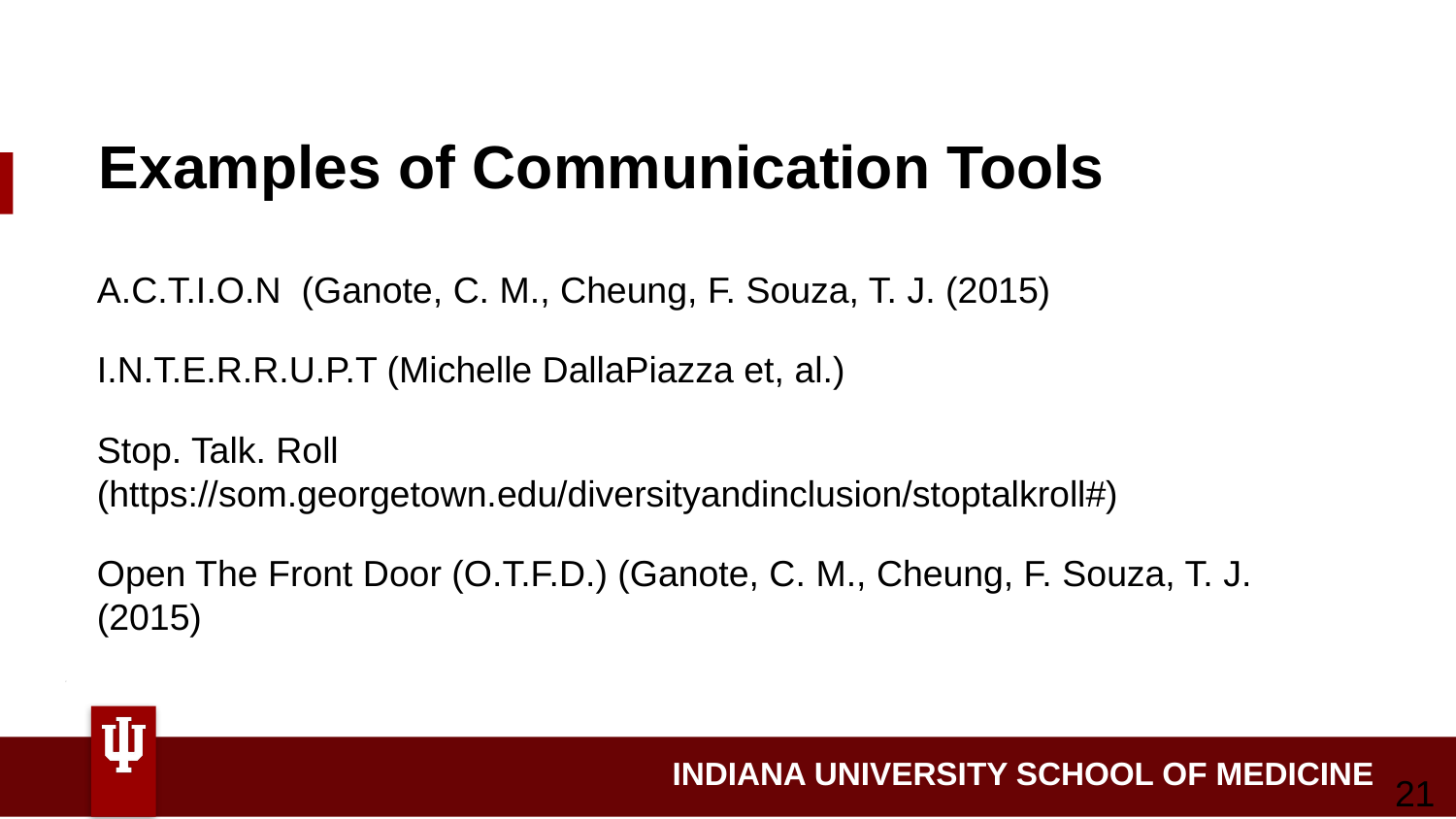

# Examples of Communication Tools
A.C.T.I.O.N (Ganote, C. M., Cheung, F. Souza, T. J. (2015)
I.N.T.E.R.R.U.P.T (Michelle DallaPiazza et, al.)
Stop. Talk. Roll (https://som.georgetown.edu/diversityandinclusion/stoptalkroll#)
Open The Front Door (O.T.F.D.) (Ganote, C. M., Cheung, F. Souza, T. J. (2015)
21

## Slide 22
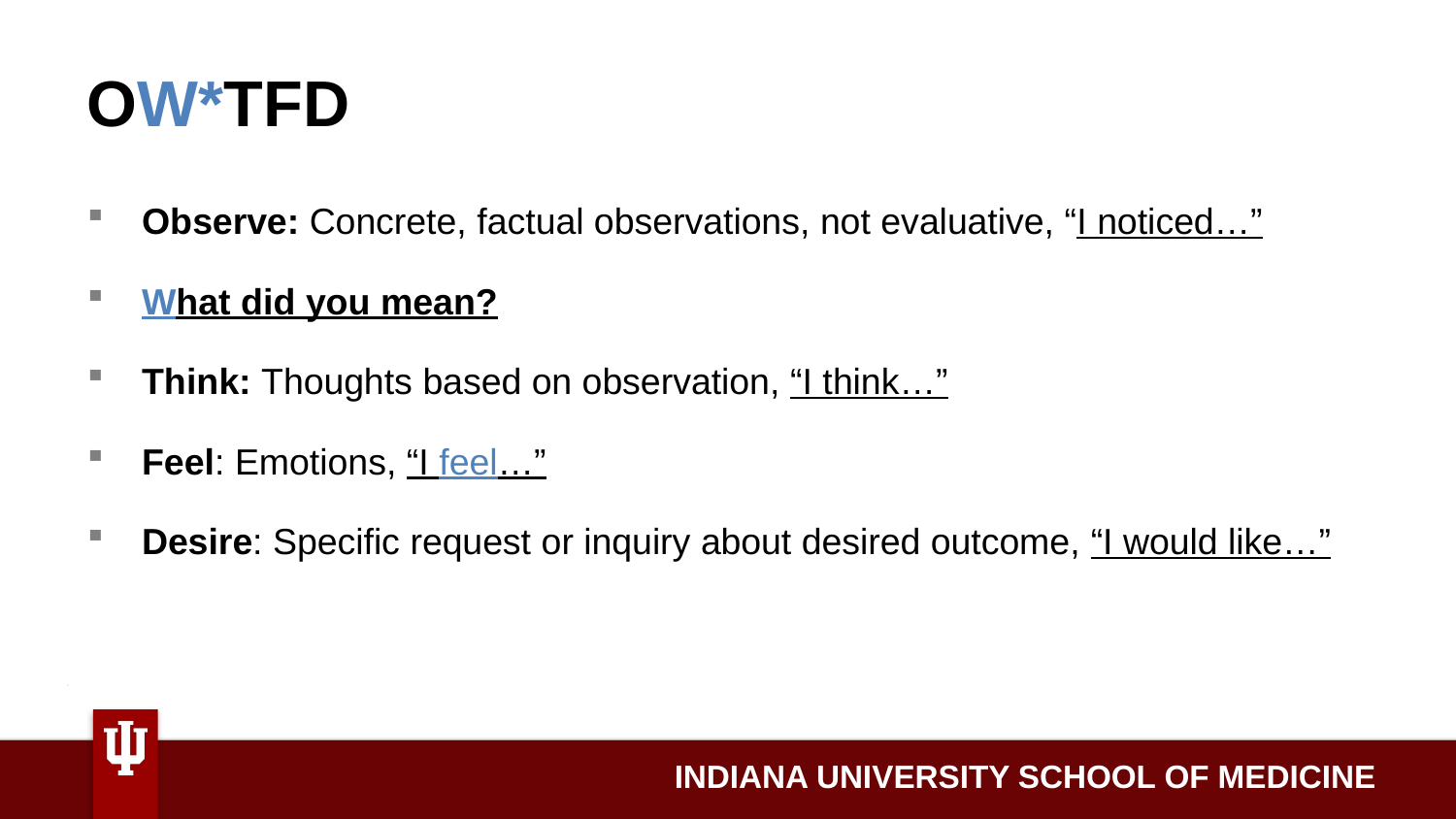

OW*TFD
Observe: Concrete, factual observations, not evaluative, “I noticed…”
What did you mean?
Think: Thoughts based on observation, “I think…”
Feel: Emotions, “I feel…”
Desire: Specific request or inquiry about desired outcome, “I would like…”

## Slide 23
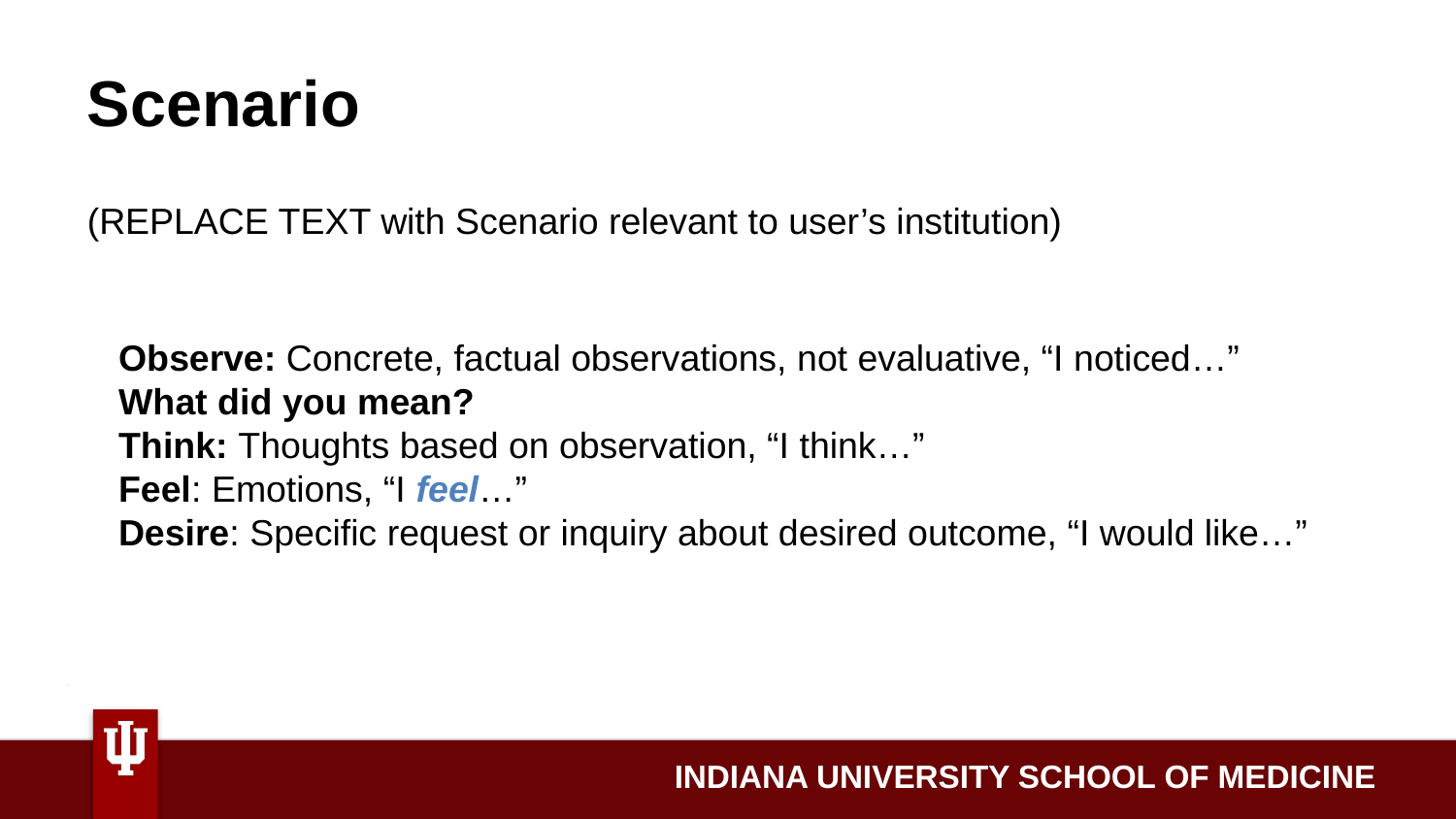

Scenario
(REPLACE TEXT with Scenario relevant to user’s institution)
Observe: Concrete, factual observations, not evaluative, “I noticed…”
What did you mean?
Think: Thoughts based on observation, “I think…”
Feel: Emotions, “I feel…”
Desire: Specific request or inquiry about desired outcome, “I would like…”

## Slide 24
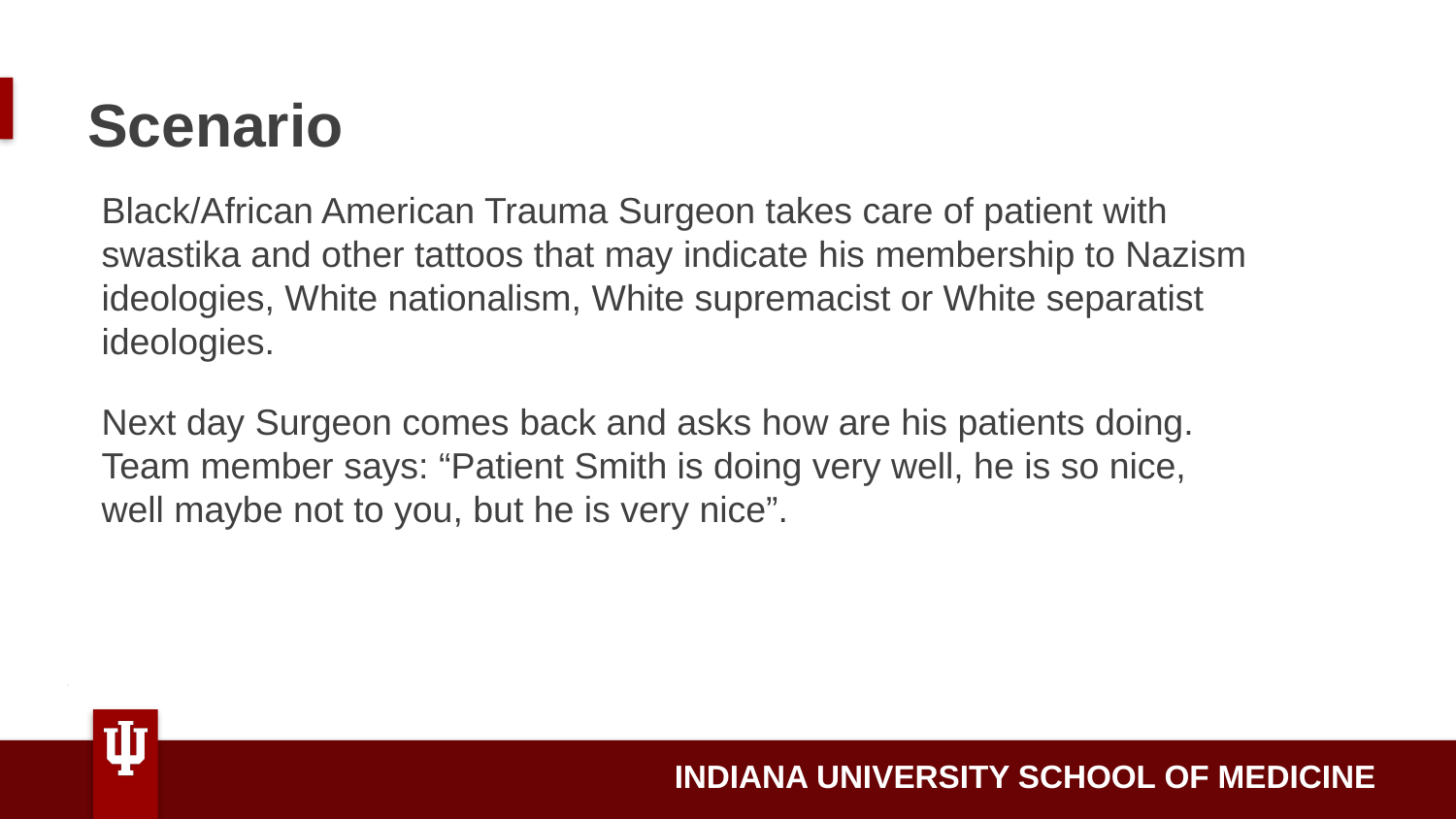

# Scenario
Black/African American Trauma Surgeon takes care of patient with swastika and other tattoos that may indicate his membership to Nazism ideologies, White nationalism, White supremacist or White separatist ideologies.
Next day Surgeon comes back and asks how are his patients doing. Team member says: “Patient Smith is doing very well, he is so nice, well maybe not to you, but he is very nice”.

## Slide 25
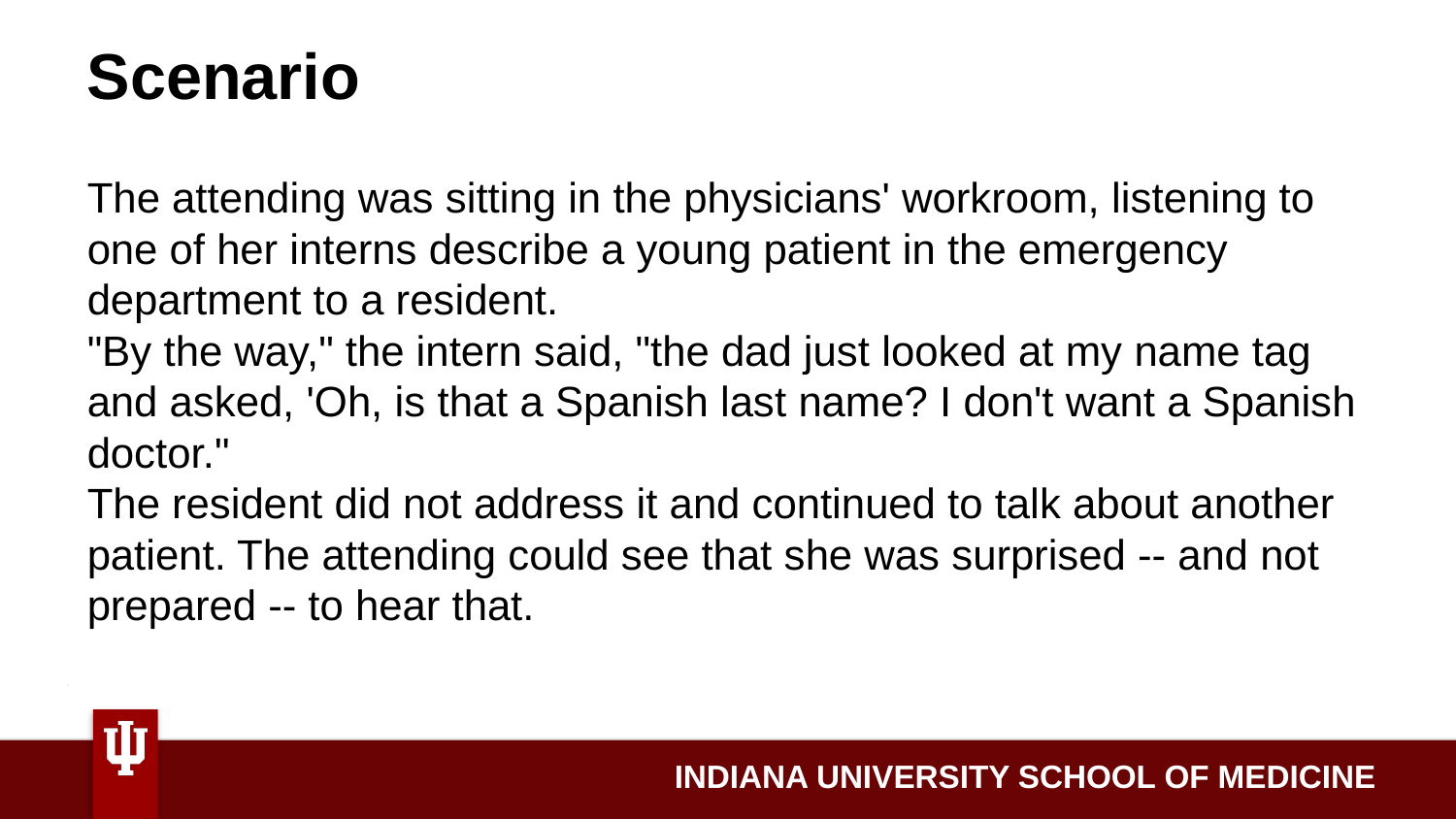

Scenario
The attending was sitting in the physicians' workroom, listening to one of her interns describe a young patient in the emergency department to a resident.
"By the way," the intern said, "the dad just looked at my name tag and asked, 'Oh, is that a Spanish last name? I don't want a Spanish doctor."
The resident did not address it and continued to talk about another patient. The attending could see that she was surprised -- and not prepared -- to hear that.

## Slide 26
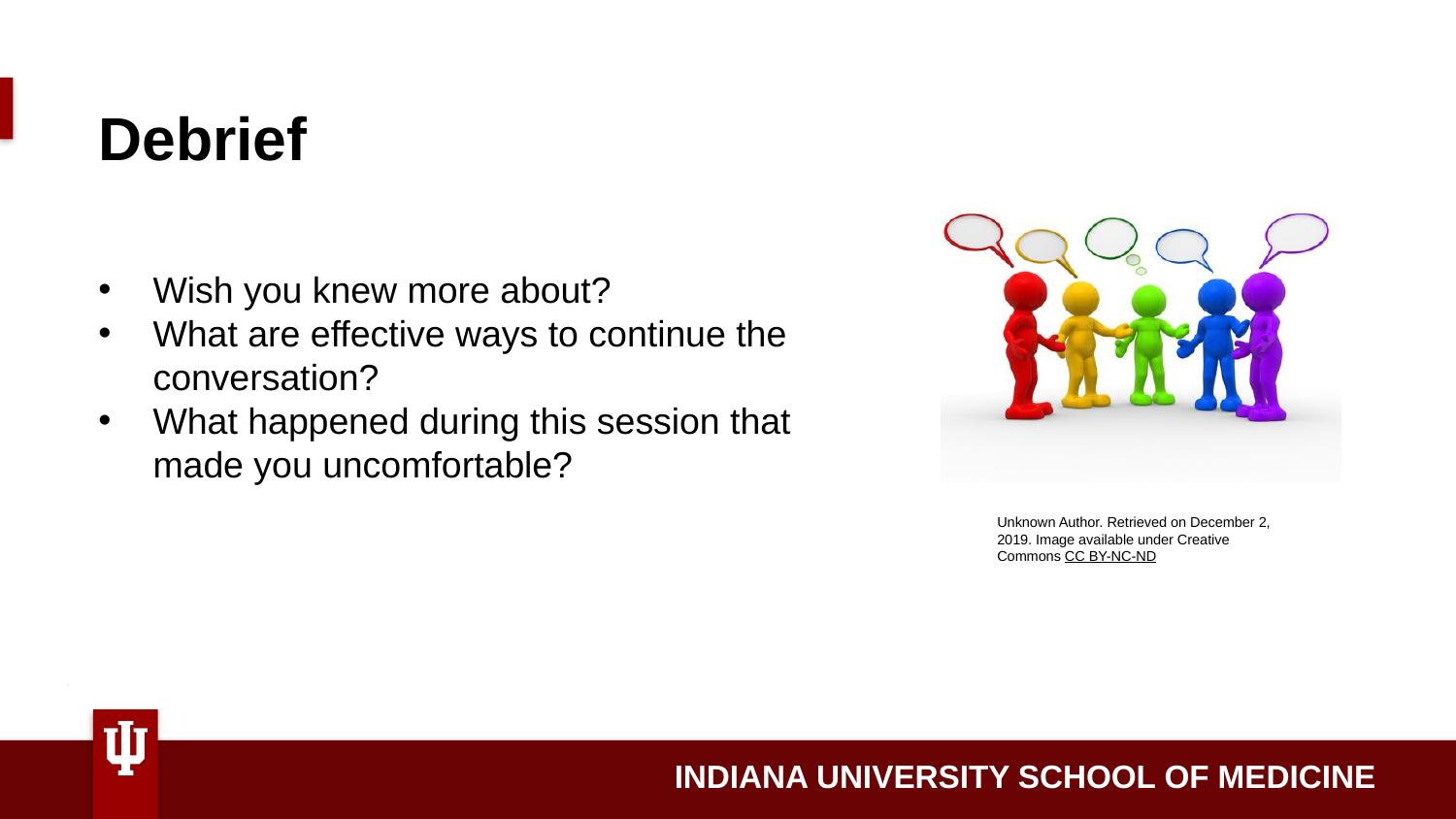

# Debrief
Wish you knew more about?
What are effective ways to continue the conversation?
What happened during this session that made you uncomfortable?
Unknown Author. Retrieved on December 2, 2019. Image available under Creative Commons CC BY-NC-ND

## Slide 27
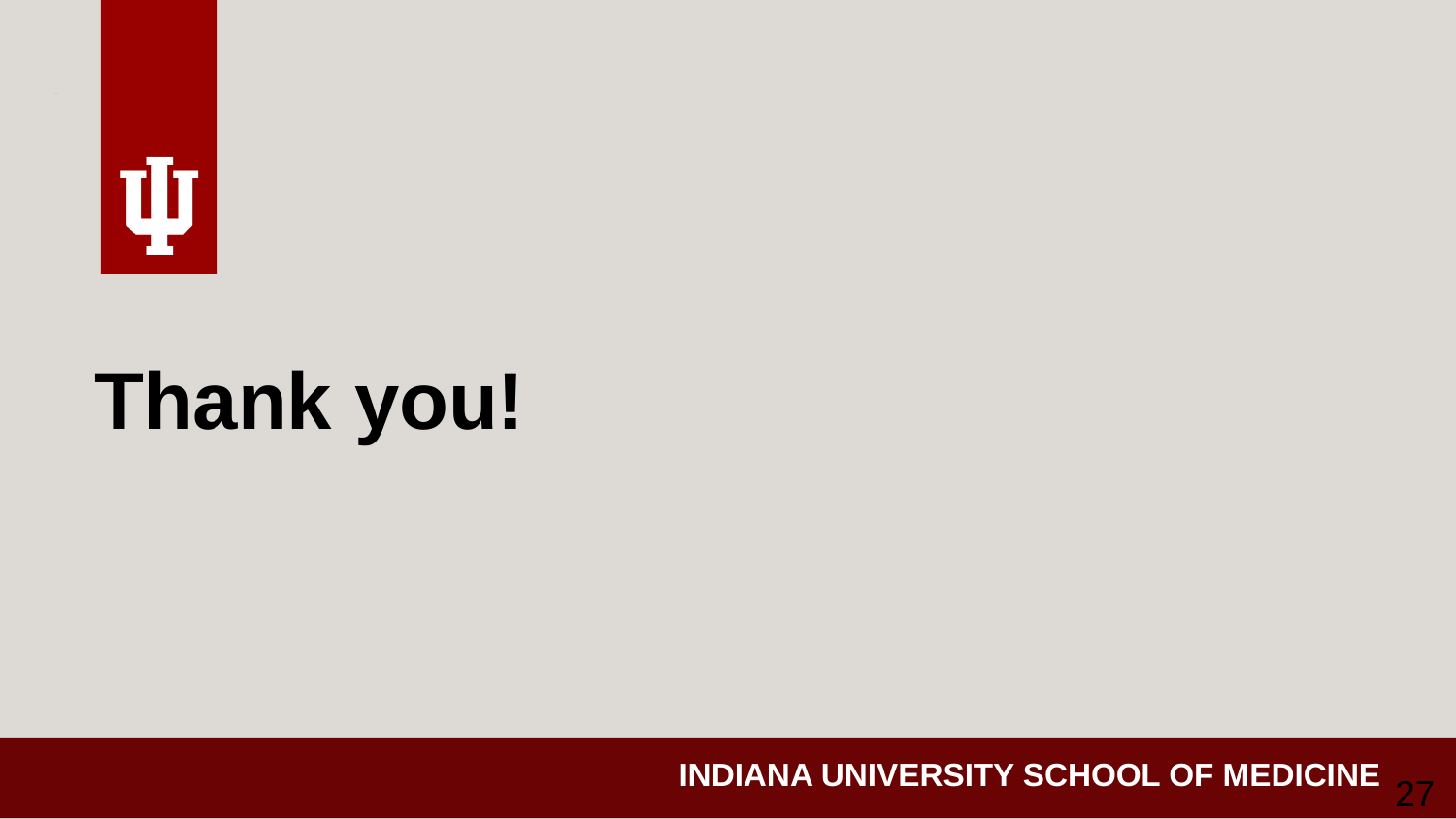

# Thank you!
27
