# Supplementary material for: “I Didn't Know What to Say”: Responding to Racism, Discrimination, and Microaggressions With the OWTFD Approach
Source: MedEdPORTAL. 2020 Jul 31;16:10971. doi: 10.15766/mep_2374-8265.10971 (PMC7394349; doi:10.15766/mep_2374-8265.10971)
Supplement: Supplementary file 1 — Workshop Agenda.docxPre- and Postsurvey.docxI Didn't Know What to Say.pptxSupplemental References.docxScenario Reenactment Script.docxScenario Guest Reflections.docxReflection Exercise.docx [file mep_2374-8265.10971-s001.zip › D. Supplemental References.docx]

**Appendix D**

**Supplemental Resources**

**Impostor Syndrome**

Bernard, D. L., Hoggard, L. S., & Neblett Jr., E. W. (2018). Racial discrimination, racial identity, and impostor phenomenon: A profile approach. *Cultural Diversity and Ethnic Minority Psychology, 24*(1), 51-61. http://dx.doi.org/10.1037/cdp0000161

Clance, P. R. & Imes, S. (1978). The imposter phenomenon in high achieving women: Dynamics and therapeutic intervention. *Psychotherapy Theory, Research and Practice, 15*(3), 1-8.

Cohen, M. J. M., Kay, A., Youakim, J. M., & Balacius, J. M. (2009). Identity transformation in medical students. *American Journal of Psychoanalysis, 69*, 43-52. DOI: 10.1057/ajp.2008.38

Day-Calder, M. (2017, June 21). Imposter syndrome. *Nursing Standard, 31*(43), 35. DOI: 10.7748/ns.31.43.35.s40

Warraich, S., Swales, C., & O’Leary, D. (2017). Thoughts of being an imposter, in medical students. *The Clinical Teacher, 14*, 454-455. DOI: 10.1111/tct.12668

**Intersectionality**

Collins, P. H. (2015). Intersectionality’s definitional dilemmas. *Annual Review of Sociology, 41*, 1-20. DOI: 10.1146/annurev-soc-073014-112142

Columbia Law School. (2017, June 8). Kimberlé Crenshaw on intersectionality, more than two decades later. Retrieved from <https://www.law.columbia.edu/pt-br/news/2017/06/kimberle-crenshaw-intersectionality>

Crenshaw, K. W. (1989). Demarginalizing the intersection of race and sex: A Black feminist critique of antidiscrimination doctrine, feminist theory and antiracist politics. *University of Chicago Legal Forum, 140*, 139-167.

Crenshaw, K. W. (1991). Mapping the margins: Intersectonality, identity politics, and violence against women of color. *Stanford Law Review, 43*(6), 1241-1299.

National Association of Independent Schools (NAIS). (2018, June 22). *Kimberlé Crenshaw: What is intersectionality?* [Video file]. Retrieved from https://www.youtube.com/watch?v=ViDtnfQ9FHc

Thomas, S. (2004, Spring). Intersectionality: The double bind of race and gender. *Perspectives.* Retrieved: <https://www.americanbar.org/content/dam/aba/publishing/perspectives_magazine/women_perspectives_Spring2004CrenshawPSP.pdf>

**Racial Battle Fatigue**

Matthews, P. [Philippe SHOCK Matthews]. (2017, May 1). *Challenging racial battle fatigue with Dr. William A. Smith* [Video file]. Retrieved from https://www.youtube.com/watch?v=f6weBHfD_Mc

Smith, W. A. (2014). Foreword. In K. J. Fasching-Varner, K. A. Albert, R. W. Mitchell, & C. Allen (Eds.), *Racial battle fatigue in higher education: Exposing the myth of post-racial America* (pp. 7-8)*.* Lanham, MD: Rowman & Littlefield Publishers.

Smith, W. A., Yosso, T. J., Solórzano, D. G. (2006). Challenging racial battle fatigue on historically White campuses: A critical race examination of race-related stress. In C. A. Stanley (Ed.), *Faculty of color: Teaching in predominately White colleges and universities* (pp. 299-327). Bolton, MA: Anker Publishing.

Smith, W. A., Allen, W. R, & Danley, L. L. (2007). “Assume the position…you fit the description”: Psychosocial experiences and racial battle fatigue among African American male college students. *American Behavioral Scientist, 51*(4), 551-578. DOI: 10.1177/0002764207307742

**Helpful video links**

[Microaggressions](https://www.youtube.com/watch?v=ZahtlxW2CIQ) in the classroom: <https://www.youtube.com/watch?v=ZahtlxW2CIQ>

How microaggressions are like mosquito bites: <https://www.youtube.com/watch?v=hDd3bzA7450>

[Grey's Anatomy Lesson](https://www.youtube.com/watch?v=JFW2cfzevio), Maggie and Amelia talk about race: <https://www.youtube.com/watch?v=xvrKrzE0nZY>
