# Supplementary material for: “I Didn't Know What to Say”: Responding to Racism, Discrimination, and Microaggressions With the OWTFD Approach
Source: MedEdPORTAL. 2020 Jul 31;16:10971. doi: 10.15766/mep_2374-8265.10971 (PMC7394349; doi:10.15766/mep_2374-8265.10971)
Supplement: Supplementary file 1 — Workshop Agenda.docxPre- and Postsurvey.docxI Didn't Know What to Say.pptxSupplemental References.docxScenario Reenactment Script.docxScenario Guest Reflections.docxReflection Exercise.docx [file mep_2374-8265.10971-s001.zip › E. Scenario Reenactment Script.docx]

**Appendix E**

**Scenario Reenactment Script**

**A note to users:** This script and reflection is provided to you to demonstrate the type of incident that was enacted during the workshop. If possible, users should identify an incident that actually occurred to someone in your community and cultivate a caring relationship to explore reenactment. If this is not possible, users may use this script to reenact the scenario.

Clinical relevance of the topic: Clinical care requires interaction with the patient, and high-quality clinical care involves the successful development of a patient-provider relationship. Bias, discrimination, and microaggressions stress these relationships. When providers are the target of such behaviors, providers often lack knowledge of how best to respond while maintaining high quality care for the patient. When medical trainees are targeted by or witness such behaviors, precepting providers must manage a complex array of patient care, clinical education, responding to and role modeling the response to the discriminatory act, and support of the trainee. The medical community will benefit from improved awareness of the prevalence of such interactions and the availability of tools to guide a response.

**Scene: Patient Room**

Setting the scene: White bearded male patient (perceived & based on record) is sitting in a chair wearing a veteran’s cap with black sunglasses and accompanied by his wife who is also white. First year fellow has just completed her chart review of the patient’s medical history and reason for clinic visit. She enters the room by knocking and being informed by the patient that she may enter the room. She is wearing her white coat and introduces herself as Dr. Duncan, Pulmonary Fellow.

**Doctor:** (knock knock)

**Patient:** Come in…

**Doctor:** Hi, my name is Dr. Duncan – I am a Pulmonary Fellow and I will be taking care of you today. (shakes hand). How are you?

**Patient:** I’m doing okay. What did you say your name was again?

**Doctor:** Dr. Duncan

**Patient:** I should take my sunglasses off so you don’t appear so black or you should turn your skin to white.

**Doctor:** Excuse me? I guess you should take your sunglasses off because I’m not turning my skin white.

**Patient:** You said your name was Duncan, right?

**Doctor:** Yes

**Patient:** You are dark skinned, so you are African right? Maybe Nigerian?

**Doctor:** No sir I was born in Georgia.

**Patient:** Is that your hair? You know black people are always changing their hair to be like white people.

**Doctor:** No sir, this is my hair. But I really don’t get why we are discussing that. Let’s focus on the reason of your visit.

**Patient:** Wait, I have more questions? What do your parents do? They must have raised you right if you were able to become a doctor.

**Doctor:** Well, my parents are both college educated and have shown me the value of a good education and hard work. That’s how I became a doctor!

**Patient:** Well, I am just curious because I haven’t seen doctors that look like you. Where did you go to college?

**Doctor:** I attended Tuskegee University in Tuskegee, Alabama.

**Patient:** That’s one of those all black schools isn’t it?

**Doctor:** It is a historically black college and university. My college is one of the top 2 producers of African American pre-medical students that go on to become doctors in this country. Now if it is alright with you, can I begin with my assessment of the reason of your visit?

After the scene: Dr. Duncan shared this experience with Dr. Joe Smith, the attending. Dr. Smith’s reflection below describes what he did in response; in the workshop, he narrates this response. There is no reenactment of Dr. Smith’s response.
