# Supplementary material for: “I Didn't Know What to Say”: Responding to Racism, Discrimination, and Microaggressions With the OWTFD Approach
Source: MedEdPORTAL. 2020 Jul 31;16:10971. doi: 10.15766/mep_2374-8265.10971 (PMC7394349; doi:10.15766/mep_2374-8265.10971)
Supplement: Supplementary file 1 — Workshop Agenda.docxPre- and Postsurvey.docxI Didn't Know What to Say.pptxSupplemental References.docxScenario Reenactment Script.docxScenario Guest Reflections.docxReflection Exercise.docx [file mep_2374-8265.10971-s001.zip › F. Scenario Guest Reflections.docx]

**“I Didn’t Know What to Say”**

Dr. Sotto: How did you come up with the script? How did you decide what to act out?

Dr. Duncan:

Being a participant in the workshop entitled “I Didn’t Know What to Say” has been an extremely rewarding experience. I say this because it has afforded me the platform to share my story, a story that I had already decided needed to be told. Storytelling is a powerful way to communicate and share experiences. Many would say that when we share stories that have really shaped our way of thinking, it can have the same effect on the listener as well. It is in that moment that the brains of the storyteller and the listener synchronize. As such, I decided the most effective way to emote my personal experience was by me becoming vulnerable and sharing a real life encounter.

This skit was created based on a real physician-patient interaction. The context of this encounter was my first day in clinic as a Pulmonary Fellow. I was eager to start the day and see patients as the next chapter of my professional career had commenced. I knocked on the door and waited for an invitation to enter the patient’s room. What lied on the other side of that door has changed my life forever – for the better. For it is out of tragedy that one learns to appreciate triumphs. I wrote this script and included a small glimpse into the physician-patient interaction I encountered that day. While some of the conversation was left out, the message was the same. This patient was racist and did not understand that his comments and questions were offensive and hurtful. As physicians, we are there to take care of others in their most vulnerable state. But does that mean that our feelings are not valued in those encounters?

While I have experienced these types of comments in my day-to-day life, and even in my medical training, it was on this day that I decided that “enough was enough”. I felt that at this point in my medical career I had “earned the right” to also be valued as a person and was going to stand up to the racial discrimination and micro-aggressions I had experienced. It was as if time stopped for a moment and I quickly thought I have three options. (1) I could get angry. I had every right to be angry, but what would that solve? (2) I could run out of the room and inform my Attending of this racist patient. But then I felt I could stand up for myself in this moment. (3) I could use this as a teaching opportunity to tell the patient his comments were offensive and be a positive example of an African American Physician and dispel the myth that “we” don’t exist or don’t “belong” in medicine. I decided to do the latter.

I thought this would be good to highlight in the skit because it would serve as an example of how to stand up for yourself in a professional manner while still providing good care to the patient.

Dr. Smith:

When Dr. Duncan entered the staffing room after her interaction with the patient, her anger and disappointment were visible despite outward professionalism. Her description of her conversation with the patient left the faculty and fellows in the room silent and in shock.

That day, I took care of the patient and did not confront him about his behavior. As I developed a formal response, I asked Dr. Duncan to meet with me to reflect on the experience and to review and approve my response. It was during this discussion that I learned much more from Dr. Duncan. I learned that this was one of many similar experiences; that in prior experiences she had not responded so eloquently; and that in those prior experiences she feared that responding could impact her ability to achieve her future career goals. I learned that Dr. Duncan called her Mother that evening after clinic, and I learned that her conversation with her Mother was emotional. Dr. Duncan approved of my formal response but expressed fear of retaliation and asked if my actions would affect her.

This powerful conversation offered me a very personal example of the impact of this patient’s behavior on Dr. Duncan, and during this same meeting we set out to find a way to raise awareness on the IU Medical Center campus.

**How was it being a part of the workshop?**

Dr. Duncan:

I had the opportunity to attend and participate in the workshop twice and each time the discussion was different. Prior to the workshop I felt very comfortable with discussing the topic of microaggressions in medical education amongst other minorities in medicine. However, I did not feel that white people were interested in the discussion. Not because they were racist, but because they think that microaggressions occur infrequently and are inconsequential in the medical training of underrepresented minorities in medicine. But this is far from the truth! I was pleasantly surprised by the response of other participants, their ability to identify microaggressions in medical education as a problem and willingness to be apart of the solution. In addition to being an attendee of the workshop, I was a participant in the skit and created questions for the Question and Answer session with Dr. Joe Smith and the audience. I found myself vulnerable each time I performed the skit and it brought up real emotions that I experienced that day which I had previously buried. I realized I made the right decision to share my story when others found comfort and the strength to stand up for themselves in the future.

Dr. Smith:

In the weeks leading up to the first workshop, I spent a great deal of time reflecting on my own decision to not confront the patient in real-time. I struggled with my decision, and I fretted that the audience would find my lack of an immediate response inadequate. Much of the time, I focused my thoughts on why I responded in such a way.

During the first workshop, I found that my honesty and vulnerability were received with respectful discussion and appreciation. As the discussion proceeded, I found that my privileged White male concerns about “saying the wrong thing” were shared by other workshop participants. The open engagement of the audience made clear to me that our campus was ready for this discussion. I also felt a great sense of pride for Dr. Duncan and her successful conversion of a very disappointing experience into an opportunity for change.
