# Supplementary material for: “I Didn't Know What to Say”: Responding to Racism, Discrimination, and Microaggressions With the OWTFD Approach
Source: MedEdPORTAL. 2020 Jul 31;16:10971. doi: 10.15766/mep_2374-8265.10971 (PMC7394349; doi:10.15766/mep_2374-8265.10971)
Supplement: Supplementary file 1 — Workshop Agenda.docxPre- and Postsurvey.docxI Didn't Know What to Say.pptxSupplemental References.docxScenario Reenactment Script.docxScenario Guest Reflections.docxReflection Exercise.docx [file mep_2374-8265.10971-s001.zip › G. Reflection Exercise.docx]

**Responding to Instances of Racism, Discrimination, and Microaggressions Reflection**

Please reflect on the following questions.

1. What motivates you to respond (or not) to instances of racism, discrimination, and/or microaggressions?
2. To what extent do you believe your role as an educator or as a staff member influences the broader learning environment for learners?
